# Human cutaneous interfollicular melanocytes differentiate temporarily under genotoxic stress

**DOI:** 10.1016/j.isci.2022.105238

**Published:** 2022-09-28

**Authors:** Per Fessé, Jan Nyman, Ingegerd Hermansson, Maj-Lis Book, Johan Ahlgren, Ingela Turesson

**Affiliations:** 1 Centre for Research and Development, Uppsala University/Region Gävleborg, Gävle, Sweden; 2 Department of Immunology, Genetics and Pathology, Experimental and Clinical Oncology, Uppsala University, Uppsala, Sweden; 3 Department of Oncology, Institute of Clinical Sciences, Sahlgrenska Academy, University of Gothenburg, Gothenburg, Sweden; 4 Department of Oncology, Faculty of Medicine and Health, Örebro University, Örebro Sweden

**Keywords:** Cancer, Cell biology, Developmental biology, Stem cells research

## Abstract

DNA-damage response of cutaneous interfollicular melanocytes to fractionated radiotherapy was investigated by immunostaining of tissue sections from punch biopsies collected before, during, and after the treatment of patients for breast cancer. Our clinical assay with sterilized hair follicles, excluded the migration of immature melanocytes from the bulge, and highlighted interfollicular melanocytes as an autonomous self-renewing population. About thirty percent are immature. Surrounding keratinocytes induced and maintained melanocyte differentiation as long as treatment was ongoing. Concomitant with differentiation, melanocytes were protected from apoptosis by transient upregulation of Bcl-2 and CXCR2. CXCR2 upregulation also indicated the instigation of premature senescence, preventing proliferation. The stem cell factor BMI1 was constitutively expressed exclusively in interfollicular melanocytes and further upregulated upon irradiation. BMI1 prevents apoptosis, terminal differentiation, and premature senescence, allowing dedifferentiation post-treatment, by suppressing the p53/p21-and p16-mediated response and upregulating CXCR2 to genotoxic damage. The pre-treatment immature subset of interfollicular melanocytes was restored after the exposure ended.

## Highlights

• Epidermal melanocytes are autonomous, self-renewing, and one-third undifferentiated
• Constitutive activity of BMI1 elicits stem cell properties of epidermal melanocytes
• BMI1 is a candidate for mediating suppression of p53/p21 and p16 upon genotoxic stress
• Epidermal melanocytes survive genotoxic exposure via temporary differentiation

## Graphical abstract

**Figure:**
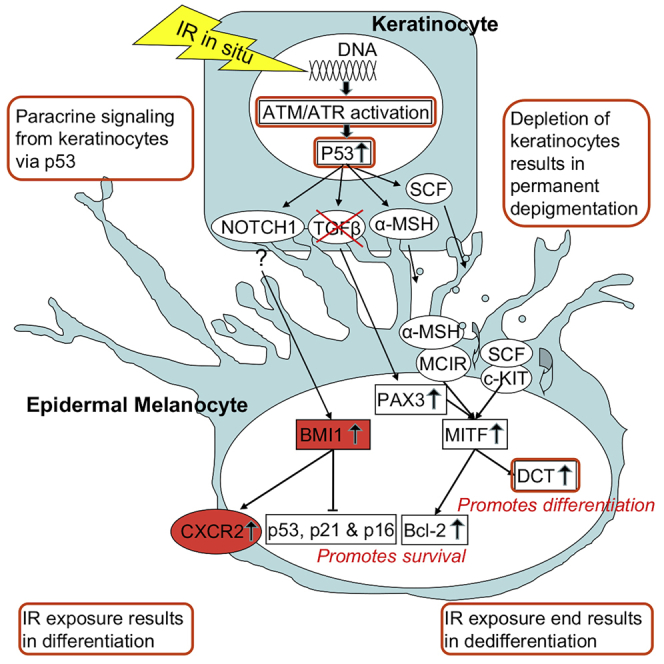

## Introduction

Cutaneous interfollicular melanocytes lie in the basal layer of the epidermis and are essential to defending the skin from the genetic insults of UV radiation. Melanin pigment synthesized in melanocytes and distributed to adjacent keratinocytes scatters UV radiation and absorbs free radicals to minimize the DNA damage that can lead to malignant transformation of keratinocytes and melanocytes and even to cell death. Melanin also protects the underlying dermis from UV damage. Each melanocyte in the basal layer is functionally related to its neighboring keratinocytes and underlying fibroblasts in the dermis.

UV radiation and ionizing radiation used for radiotherapy cause base damage and single-strand breaks in DNA. The most serious genetic insults, DNA double-strand breaks, occur much more often after ionizing radiation (Blackford and Jackson, 2017; Hustedt and Durocher, 2016; Sarkar and Gaddameedhi, 2020; Vignard et al., 2013). Compared with the current understanding of the molecular signaling induced by UV radiation, the melanocyte response to ionizing radiation is poorly characterized.

Melanocytes arise from neural crest cells, which include melanoblasts, the precursors of melanocytes. Melanoblasts are derived from a bipotential glial-melanocyte lineage progenitor (Liu et al., 2014). During embryonic development, melanoblasts migrate through the dermis and cross the basement membrane at the border between the dermis and epidermis to colonize the basal cell layer and hair follicles. In humans, immature melanocytes also remain in the interfollicular epidermis. Epidermal melanocytes are responsible for tanning and protection against UV radiation. The bulge of the hair follicle is the niche that creates and harbors melanocyte stem cells in adults. Bulge stem cells are quiescent and considered to be a reservoir involved in each hair cycle, and also supply interfollicular melanocytes as needed during adulthood (Bertrand et al., 2020).

Recently, we described the response of cutaneous interfollicular melanocytes in the clinical setting of fractionated radiotherapy, with daily subtherapeutic doses in the range of 0.05-1.1 Gy applied for 7 weeks (Fesse et al., 2019). In that work, we identified a subset of immature interfollicular melanocytes that exhibit hypersensitivity to differentiation in the very low dose region, manifested at 1 week of radiotherapy and confirmed by the end of 7 weeks of treatment. However, the behavior of the melanocytes after the completion of radiotherapy was not investigated.

The aim of the present study was to assess the melanocyte response to the most conventional radiotherapy regimen prescribed for subclinical tumor disease (i.e., daily dose fractions of 2.0 Gy applied for 5 weeks) by weekly assessments during the radiotherapy course and also up to 5 weeks post-treatment. We analyzed the expressions of molecular markers involved in survival, differentiation, proliferation, apoptosis, and premature senescence. The findings suggest that epidermal interfollicular melanocytes *in situ* are an autonomous, self-renewing population exhibiting various degrees of differentiation and with absolute radio-resistance to cell death because of stem cell properties. Epidermal melanocytes respond with transient differentiation to genotoxic stress.

## Results

### Identification of epidermal melanocytes

Epidermal melanocytes are easily identified by their morphological characteristics: attachment to the basement membrane, nucleus-adherent cytoplasm, and lack of desmosomes (Barlow et al., 2007; Lin and Fisher, 2007). Here, microscopic counting at 1000× magnification was performed to distinguish melanocytes from keratinocytes morphologically, as keratinocytes display desmosomes in the cell membrane at this high power; the keratinocytes’ desmosomes cannot be seen at 400× magnification.

**Figure 1:**
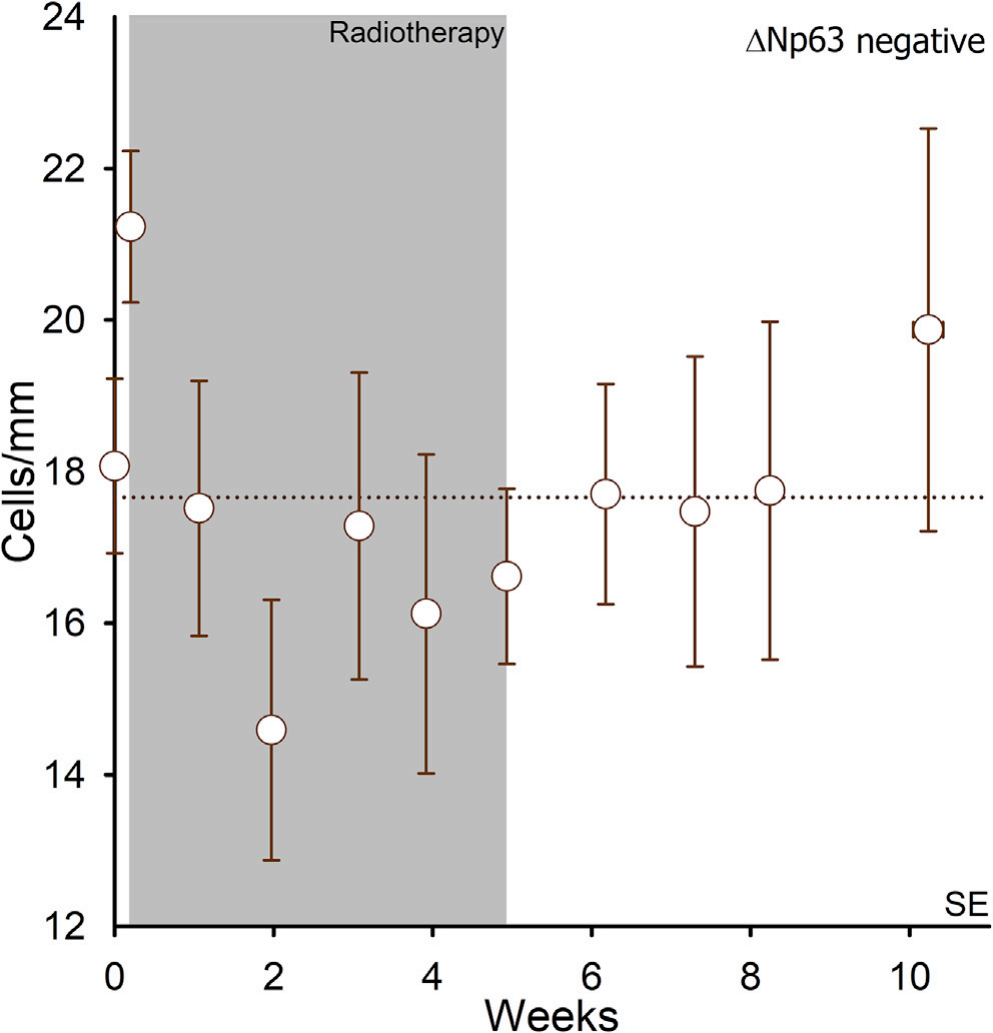
The number of melanocytes is undisturbed following the radiotherapy course Mean number of ΔNp63-negative () cells per millimeter in the basal layer of epidermis (n = 15 patients). Error bars represent SEM. The dashed line represents the average of the data points.

Because ΔNp63 is a cell cycle regulator expressed in keratinocytes but undetectable in normal melanocytes (Kulesz-Martin et al., 2005), we used this marker to distinguish melanocytes from keratinocytes, characterizing the two cell types by immunostaining. All ΔNp63-negative cells matched the morphological criteria for epidermal melanocytes; in this staining, totally 16600 epidermal melanocytes were counted and presented as cells/mm of the basement membrane at each time point. In unexposed skin, the ΔNp63-negative cell number was 18.1 ± 1.2 cells/mm (Table 1, Figure 1); at 30 min after the first 2-Gy fraction a somewhat higher number was determined (p < 0.01), and at 2 weeks of radiotherapy a reduced cell number was observed (p < 0.05), but did not otherwise significantly fluctuate during or after radiotherapy. After 5 weeks of treatment, there were 16.6 ± 1.2 cells/mm, which was near unexposed skin. No time-response relationship could be demonstrated post-treatment (p = 0.1). Given these features, we considered the number of ΔNp63-negative cells to be undisturbed through the treatment and up to 5 weeks after its cessation (see Comments in STAR methods).

**Table 1:** Mean number of cells per millimeter for each marker

Staining	Time in weeks | *number of biopsies* | number of patients										
	0|*30*|15	0.2|*13*|13	1|*19*|13	2|*13*|10	3|*10*|8	4|*9*|5	5|*15|*15	6|*13*|13	7|*9*|9	8|*7*|7	10|*3*|3
ΔNp63 negative	18.1 (1.2)	21.2 (1.0)	17.5 (1.7)	14.6 (1.7)	17.3 (2.0)	16.1 (2.1)	16.6 (1.2)	17.7 (1.5)	17.5 (2.1)	17.8 (2.2)	19.9 (2.7)
MITF positive	14.1 (1.5)	14.8 (1.5)	17.6 (1.6)	18.2 (1.8)	20.5 (1.7)	18.7 (2.7)	20.8 (1.5)	21.5 (1.6)	19.4 (2.2)	20.4 (3.3)	15.4 (2.8)
MITF negative	5.2 (0.3)	5.7 (0.4)	1.9 (0.3)	1.1 (0.2)	0.8 (0.2)	1.1 (0.3)	1.0 (0.1)	1.9 (0.2)	2.8 (0.6)	3.5 (0.7)	6.6 (0.4)
Bcl-2 positive	16.9 (1.2)	19.3 (1.7)	20.2 (1.6)	21.0 (1.8)	23.4 (1.5)	22.8 (2.8)	23.3 (1.1)	22.5 (1.6)	21.8 (1.8)	21.5 (2.8)	19.6 (1.4)
Bcl-2 negative	4.0 (0.4)	3.8 (0.3)	1.3 (0.2)	0.7 (0.1)	0.6 (0.1)	0.8 (0.3)	0.7 (0.1)	0.9 (0.2)	1.5 (0.2)	2.1 (0.6)	4.8 (0.7)
BMI1 positive	15.9 (0.9)	17.0 (0.9)	16.7 (1.3)	15.9 (1.3)	16.8 (1.4)	18.4 (3.0)	17.4 (1.0)	18.3 (1.2)	17.7 (1.6)	17.3 (1.6)	20.9 (1.4)
BMI1 negative	2.5 (0.3)	1.9 (0.4)	0.6 (0.1)	0.4 (0.1)	0.4 (0.1)	0.2 (0.1)	0.4 (0.1)	0.7 (0.1)	1.3 (0.4)	1.4 (0.7)	1.8 (0.2)
pRb positive	0.03 (0.01)	0.04 (0.02)	0.01 (0.01)	0.01 (0.01)	0.02 (0.02)	0 (0)	0 (0)	0 (0)	0.06 (0.03)	0 (0)	0 (0)
p21-positive nucleus	0.09 (0.03)	0.05 (0.03)	0.4 (0.08)	0.4 (0.09)	0.6 (0.2)	0.6 (0.2)	0.7 (0.1)	0.3 (0.05)	0.3 (0.1)	0.2 (0.04)	0.2 (0.1)
p21-positive cytoplasm	0.7 (0.2)	0.6 (0.2)	1.9 (0.4)	2.1 (0.5)	2.4 (0.9)	2.9 (1.4)	2.9 (0.5)	1.6 (0.3)	1.4 (0.4)	1.1 (0.3)	1.4 (0.3)

Data are presented as mean and SEM. CV in the control for ΔNp63-negative 25%, MITF-positive 29%, MITF-negative 24%, Bcl-2-positive 28%, Bcl-2-negative 36%, BMI1-positive 21% and BMI1-negative 45%.

The individual differences in the number of melanocytes described by the coefficient of variation were 25% for the ΔNp63-negative cells. Therefore, a biopsy series from each patient, allowing an individual dose response for each patient to be tracked for ΔNp63-negative cells and for each marker presented later in results, was a prerequisite to reveal dose- and time-response relationships of the epidermal melanocytes accurately enough. Importantly, the radiotherapy dose applied led to the sterilization of hair follicles (Lawenda et al., 2004), so there was no migration of melanocyte stem cells from the hair bulge to the interfollicular epidermis.

### Differentiation and anti-apoptotic response to ionizing radiation

**Figure 2:**
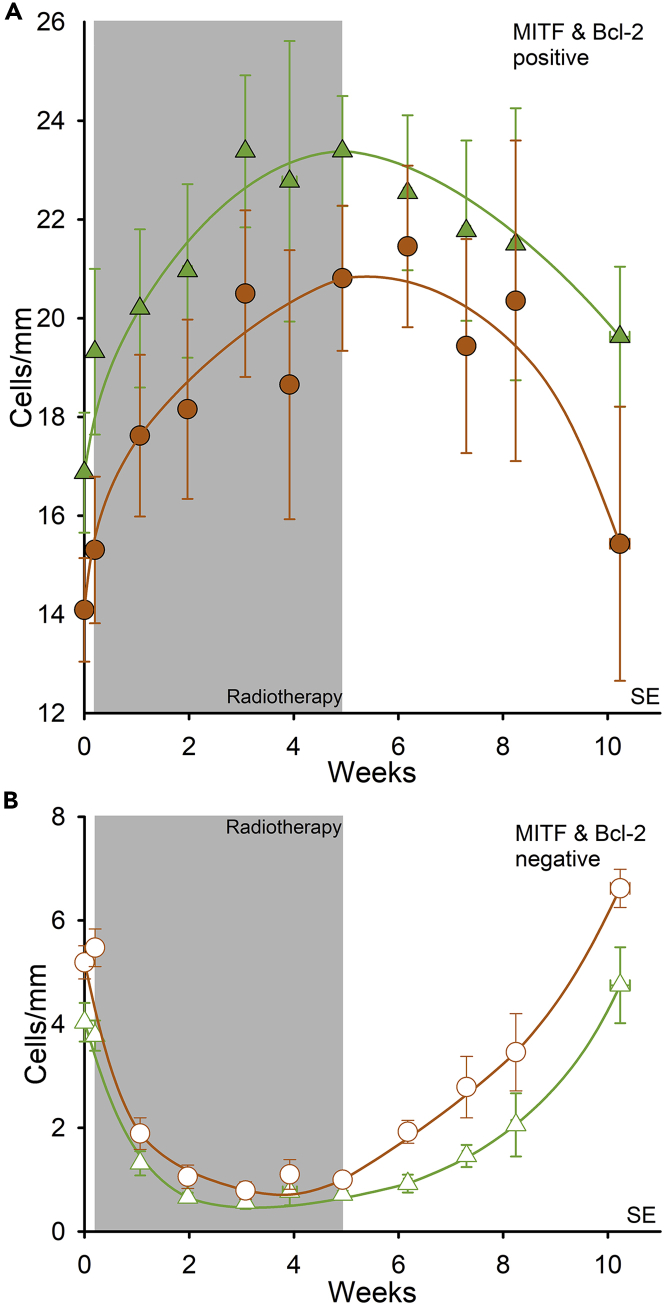
A subpopulation of immature melanocytes exists in the interfollicular epidermis that differentiates temporarily during radiation exposure Mean number of cells per millimeter in the basal layer (n = 15 patients). (A) MITF-positive melanocytes () and Bcl-2-positive melanocytes (). (B) MITF-negative cells () and Bcl-2-negative cells () are morphologically characterized as melanocytes. Error bars represent SEM.

Microphthalmia transcription factor (MITF) is a melanocyte-specific protein (Hodgkinson et al., 1993) and the master regulator of melanocyte function (Goding and Arnheiter, 2019). MITF regulates melanin synthesis and melanocyte survival, differentiation, and proliferation (Kubic et al., 2008). Using an anti-MITF antibody, we assessed MITF expression in total 23160 interfollicular melanocytes, presented in cells/mm of the basement membrane at each time point. We found distinct staining in the melanocyte nucleus. The number of MITF-stained cells was 14.1 ± 1.5 cells/mm in unexposed skin and was unchanged 30 min after the first 2-Gy fraction (p = 0.60) (Table 1, Figure 2A). Throughout radiotherapy, the number of MITF-stained cells increased successively up to the end of 5 weeks (p < 0.001), reaching 20.8 cells/mm. From 1 to 3 weeks after the completion of radiotherapy, the number of MITF-stained cells was still high compared to pre-treatment. However, at 5 weeks post-treatment, the number of MITF-positive cells had declined to 15.4 ± 2.8 cells/mm and did not differ from that of unexposed skin.

All MITF-positive cells in the basal layer exhibited the morphological characteristics of melanocytes, but some MITF-negative cells also had typical melanocyte morphology. We assessed this subset separately. The number of MITF-negative melanocytes was 5.2 ± 0.3 cells/mm in unexposed skin, which was unchanged at 30 min after the first treatment of 2 Gy (p = 0.20) (Table 1). At 1 week of radiotherapy, we observed a significant decrease in MITF-negative melanocytes to 1.9 ± 0.3 cells/mm (p < 0.001); over the next 4 weeks the number remained significantly lower, at 0.8-1 cells/mm, compared with unexposed skin (p < 0.001). We saw this reduction in MITF-negative melanocytes in every patient during treatment (presented in the supplement). After treatment completion, the number of MITF-negative cells increased steadily (p < 0.001). At 5 weeks post-treatment, the number reached 6.6 ± 0.4 cells/mm, which was close to the pre-treatment number.

Double staining with MITF and ΔNp63 provided reassurance that MITF was expressed only in ΔNp63-negative cells, in both unexposed skin and after radiotherapy (Figure S1A). Of note, in unexposed skin, approximately one-third of ΔNp63-negative cells were MITF-negative, 86 of a total of 235 epidermal melanocytes (36.6%). Radiotherapy resulted in an increase in MITF-positive cells and a concomitant significant decrease in the number of cells negative for both MITF and ΔNp63 (p < 0.01). At the end of 5 weeks of treatment, the majority of ΔNp63-negative cells expressed MITF; then only 42 of the total 240 melanocytes (17.5%) were still MITF-negative. Of note, at 5 weeks post-treatment, the MITF-negative cells increased significantly (p < 0.01), then 94 of total 257 epidermal melanocytes (36.6%) were MITF-negative, equal to the pre-treatment number.

MITF upregulates s the anti-apoptotic protein Bcl-2 (McGill et al., 2002), leading us to assess Bcl-2 expression in totally 21380 interfollicular melanocytes, presented as cells/mm of the basement membrane at each time point. We observed Bcl-2 protein only in the melanocyte cytoplasm. Bcl-2 expression is much lower or undetectable in keratinocytes compared with melanocytes in the epidermis (Bowen et al., 2003), and we confirmed by morphology that Bcl-2 staining was restricted to melanocytes. The number of Bcl-2-stained cells was estimated to be 16.9 ± 1.2 cells/mm in unexposed skin (Table 1, Figure 2A); at 30 min after the first 2-Gy fraction a somewhat higher count was noticed (p < 0.01). During the subsequent 5 weeks of radiotherapy, positive cell counts significantly increased (p < 0.001), reaching 23.3 ± 1.1 cells/mm. Post-treatment, the number of Bcl-2-stained cells decreased but remained higher than the pre-treatment number for at least 5 weeks (p < 0.01).

Similar to our finding of a subpopulation of MITF-negative melanocytes, we identified cells that did not stain for Bcl-2 but that matched the morphological characteristics of melanocytes. The number of Bcl-2-negative cells was 4.0 ± 0.4 cells/mm in unexposed skin, which was unchanged at 30 min after the first treatment of 2 Gy (p = 0.50) (Table 1, Figure 2B). At 1 week of radiotherapy, we observed a significant decrease in Bcl-2-negative cells (p < 0.001), 1.3 ± 0.2 cells/mm; over the next 4 weeks the number remained in the range of 0.7 ± 0.1 cells/mm. We saw a reduction in Bcl-2-negative cells in every patient (presented in the supplement). After patients completed radiotherapy, the number of Bcl-2-negative cells started to increase (p < 0.001). At 5 weeks post-treatment, the number reached 4.8 ± 0.7 cells/mm, which was similar to that of unexposed skin.

Double staining with Bcl-2 and ΔNp63 confirmed that Bcl-2 was expressed only in ΔNp63-negative cells both in unexposed skin and upon irradiation (Figure S1B). Of note, in unexposed skin, some ΔNp63-negative cells also were negative for Bcl-2, 126 of total 239 epidermal melanocytes (52.7%). Radiotherapy resulted in an increase in Bcl-2-positive cells and a concomitant significant decrease in the number of cells negative for both Bcl-2 and ΔNp63 (p < 0.001). At the end of 5 weeks of treatment, 39 of total 148 melanocytes (26.4%) were Bcl-2-negative. Thereafter, the number of Bcl-2-negative cells increased significantly (p < 0.001); 153 of totally 254 epidermal melanocytes (60.3%) were Bcl-2-negative at 5 weeks post-treatment.

It has been suggested that the upregulation of Bcl-2 by MITF regulates melanocyte survival and resistance to genotoxic stress (McGill et al., 2002). In keeping with this prediction, double staining for MITF and Bcl-2 proved that all MITF-positive cells were also Bcl-2-positive in our previous study (Fesse et al., 2019). This also should be true for the present cohort. To illustrate the evaluation of immunohistochemical staining, the individual dose-responses of MITF and Bcl-2 were presented for positive cells (Figure S2) and negative cells (Figure S3), illustrating a close relationship between the two markers; this was confirmed by the unequivocal correlation between Bcl-2 and MITF for each individual patient in the whole cohort at all time points (p < 0.001) (Figure S4).

Furthermore, we quantified the expression of SOX10 to assess the initiation of melanocyte differentiation following radiotherapy-induced injury. SOX10 is a transcription factor expressed in neural crest cells and is crucial for commitment to the melanocyte lineage. It also is expressed in progenitor cells and early differentiated melanocytes (Harris et al., 2013; Shakhova et al., 2015). Nuclear SOX10 is visible by immunohistochemical staining in a subset of normal human melanocytes in the basal layer of the epidermis (Nonaka et al., 2008). Upon exposure to UV radiation, SOX10 is necessary for the initiation of melanocyte differentiation and melanin synthesis.

In tissue sections stained for SOX10, from five patients, all SOX10-positive cells in the basal layer had melanocyte morphology; in total 2 490 interfollicular melanocytes were counted and presented as cells/mm of the basement membrane at the different time points. The number of SOX10-stained cells was estimated to be 15.0 ± 2.4 cells/mm in unexposed skin, increasing to 18.7 ± 2.4 cells/mm (p = 0.17) at the end of the 5-week treatment period. The counts then declined to 16.3 ± 6.1 cells/mm during the first 3 weeks post-treatment, similar to that in unexposed skin.

In addition to the MITF- and Bcl-2-negative subpopulation of melanocytes, we identified cells that did not stain for SOX10 but had melanocyte morphology. The number of SOX10-negative cells in unexposed skin was 6.7 ± 0.6 cells/mm. At 5 weeks of radiotherapy, we observed a significantly lower number of SOX10-negative cells (p < 0.01), 1.6 ± 0.3 cells/mm. By 3 weeks post-treatment, the number had increased again to 5.4 ± 0.1 cells/mm, which was not different from unexposed skin. Determinations of the proportions of immature melanocytes before, at end of treatment, and post-treatment in the staining for SOX10, MITF, and Bcl-2 are summarized in Table 2.

**Table 2:** Number of negative epidermal melanocytes per millimeter and percentage of the total number melanocytes counted for each marker

Staining	Control		End of RT		Post RT	
	Cells/mm	%	Cells/mm	%	Cells/mm	%
SOX10	6.7 ± 0.6	32.2 ± 0.03	1.6 ± 0.3	7.6 ± 0.01	5.4 ± 0.1	27.3 ± 0.08
MITF	5.2 ± 0.3	28.1 ± 0.02	1.0 ± 0.1	5.1 ± 0.01	6.6 ± 0.4	30.9 ± 0.03
Bcl-2	4.0 ± 0.4	20.2 ± 0.02	0.7 ± 0.1	3.0 ± 0.01	4.8 ± 0.7	19.3 ± 0.02

Data are presented as mean and SEM.

Melanocytes in the interfollicular epidermis present with varying degrees of differentiation (He et al., 2010; Medic and Ziman, 2010). Dopachrome tautomerase (DCT), one of the three enzymes activated by MITF for melanin synthesis, is a marker of early differentiation (Lang et al., 2005). In our assessment of immunofluorescence of the double staining for ΔNp63 and DCT, we observed no ΔNp63-positive cells in unexposed or radiotherapy-exposed skin that co-expressed DCT (Figure S1C). In the unexposed skin, more than half of the ΔNp63-negative cells were negative for DCT, 126 of total 224 epidermal melanocytes (56.3%). We observed a decrease in the proportion of DCT-negative cells at the end of the treatment period (p = 0.05), then 98 of total 242 (38.4%) were still DCT-negative; the DCT-positive cells also had developed increasing numbers of dendrites. At 5 weeks post-treatment, the proportion of DCT-negative epidermal melanocytes had increased significantly again (p = 0.02), up to 133 of total 225 (59.1%). However, the increase in dendrite numbers persisted.

### p53/p21 signaling is pronounced in keratinocytes upon irradiation but suppressed in adjacent melanocytes

Upon exposure to UV or ionizing radiation, epidermal keratinocytes strongly express both p53 and p21 (Kulesz-Martin et al., 2005; Turesson et al., 2001), but no or very weak expression is observed in interfollicular melanocytes *in situ* by immunostaining (Fesse et al., 2019). Although p21 mRNA and protein levels increase after DNA damage in melanocytes *in situ*, p53 binding to the promoter of p21 is not detectable (Kulesz-Martin et al., 2005), which confirms that the p53-p21 pathway is disconnected.

Our immunostaining for p53 and p21 was as expected: negative for all melanocytes and keratinocytes in unexposed skin. In contrast to the pronounced nuclear staining of keratinocytes induced by radiotherapy, nuclear staining for the p53 and p21 proteins was not obvious in melanocytes (Figure S5).

In the immunostaining for p21, totally 13,370 epidermal melanocytes were counted and presented as cells/mm of the basement membrane at each time point. During the 10 weeks of assessment, intense nuclear expression of p21 appeared in only a few melanocytes, less than 1 cell/mm. Expression of p21 in the cytoplasm was observed more frequently and peaked at 4 to 5 weeks of treatment, at 2.9 ± 0.5 cells/mm (p < 0.001; Table 1).

### Stem cell characteristics of cutaneous interfollicular melanocytes

B lymphoma Mo-MLV insertion region 1 (BMI1) is, among other things, a stem cell factor required for the self-renewal of adult neural stem cells and early progenitors (Molofsky et al., 2003). Similar to neural stem cells, melanocytes are derived from the neural crest (Sommer, 2011), but the existence and role of BMI1 expression in melanocytes have not yet been reported. BMI1 is part of polycomb repressive complex 1, which inhibits gene expression through histone modification and chromatin compaction. BMI1 can monoubiquitinate histone H2A through its E3 ligase activity via the catalytic RING1A/RING1B subunits (Francis et al., 2004; Valk-Lingbeek et al., 2004; Wang et al., 2004). In this way, BMI1 represses the INK4A/ARF gene locus, which encodes the p16INK4A and p14ARF tumor suppressors that function in the pRb and p53 pathways, respectively. The result is the regulation of senescence and cell proliferation (Jacobs et al., 1999; Lowe et al., 2004; Serrano et al., 1996; Sharpless and Sherr, 2015).

BMI1 also can directly bind to p53 in a complex with RING1A or RING1B, leading to ubiquitination and proteasomal degradation of the p53 protein in neural progenitor cells. Thus, in certain contexts, BMI1 can protect cells from both apoptosis and terminal differentiation (Calao et al., 2013; Koifman et al., 2019). This regulatory effect of BMI1 on the p53 stress response also may be relevant for interfollicular melanocytes, which do not express detectable amounts of p53 protein after UV or ionizing irradiation of human skin *in situ* (Fesse et al., 2019; Kulesz-Martin et al., 2005), and also confirmed in the present study. Of note, BMI1 is not expressed in normal human melanocytes *in vitro* (Basu et al., 2019), which may explain the upregulation of p53 protein upon UV radiation under this condition (Medrano et al., 1995).

**Figure 3:**
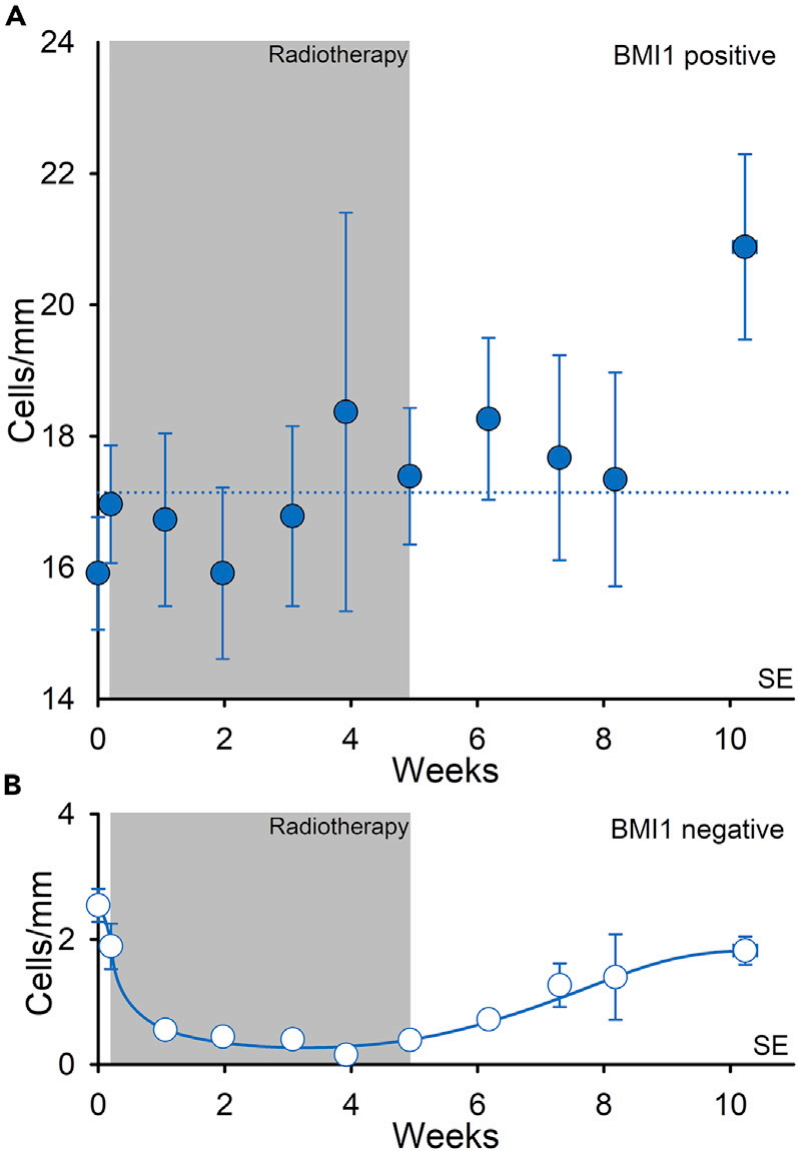
Interfollicular melanocytes express BMI1 Mean number of stained cells per millimeter in the basal layer for 15 patients. (A) BMI1-positive cells () and (B) BMI1-negative cells morphologically characterized as melanocytes (). Error bars represent SEM. The dashed line represents the average of the data points.

Independent of its suppression of the p14ARF/p53/p21 pathway, BMI1 can repress the cell cycle inhibitor p21 in neural stem cells (Fasano et al., 2007), inactivating p21 by binding directly to its promoter, as exemplified in cerebellar progenitor cells (Subkhankulova et al., 2010). In addition, BMI1 can mediate resistance to apoptosis by activating NF-kappa/β, as demonstrated for glioma cells (Li et al., 2010), except by the degradation of the p53 protein. We assessed double staining for BMI1 and ΔNp63 (Figure S6) as well as for BMI1 and SOX10 (not shown), and found that BMI1 was expressed only in melanocytes and not detectable in keratinocytes. In the immunostaining for BMI1, in total 15,390 epidermal melanocytes were counted and presented as cells/mm of the basement membrane at each time point. Almost all melanocytes in both unexposed and exposed skin expressed nuclear BMI1. A very small fraction of melanocytes in unexposed skin (2.5 ± 0.3 cells/mm) were negative for BMI1 in immunohistochemistry, yet a significant decrease was observed during radiotherapy (p < 0.001), with only 0.4 ± 0.1 cells/mm found at the completion of the treatment course (Table 1 and Figure 3, and Figure S5). Thereafter, the number of BMI1-negative melanocytes gradually increased again (p < 0.001), reaching 1.8 ± 0.2 cells/mm at 5 weeks post-treatment, equal to unexposed skin (p = 0.36). Of note, we observed BMI1-stained melanocytes also in the hair follicle bulge.

From the individual dose responses of MITF and BMI1, presented both for positive cells and negative cells (Figures S7 and S8), an obvious relationship between the two markers was noticed. This was confirmed by the strong individual correlation between MITF-positive cells and BMI1-positive cells/mm in both unexposed and exposed skin, as well as post-treatment (0-7 weeks: p ≤ 0.002; 8-10 weeks: p = 0.08; Figure S9). In addition, for the five patients with skin sections stained for both SOX10 and BMI1, we found a good individual correlation between the two markers in both unexposed and irradiated skin (p = 0.04).

Expression of BMI1 is expected to be associated with suppression of the p16 protein. In the immunostaining for p16, we could not notice any obvious nuclear expression of p16 in the epidermal melanocytes pre-treatment or at the treatment end. This is in contrast to finding that p16 is expressed in melanocytes upon UV radiation *in vitro* (Piepkorn, 2000).

Thus, protein expression of p53, p21, and p16 was demonstrated in skin melanocytes *in vitro* upon UV-radiation, but not upon genotoxic exposure *in situ* from UV and ionizing radiation. This reinforces that the BMI1expression in epidermal melanocytes regulates the suppression of all three proteins and that BMI1is governed by paracrine signaling from the adjacent keratinocytes, resulting in an effective safeguard for the survival of epidermal melanocytes.

For the consistency in the determinations of the total number of epidermal melanocytes for MITF, Bcl-2, and BMI1, and for the accuracy of quantification in immunohistochemistry and immunofluorescence, see Comments in STAR methods.

### Absence of apoptosis but the indication of a pro-senescence response of interfollicular melanocytes during the irradiation period

Apoptosis is usually assessed by the TUNEL assay. However, in our hands, this staining was not good enough for accurate quantification of apoptotic cells in skin tissue sections. Therefore, we used confluent nuclear staining with gamma-H2AX verified as a true indicator of apoptosis (Qvarnstrom et al., 2009). For the current patient cohort apoptosis was previously evaluated (Turesson et al., 2020), and very few apoptotic cells were detected. Keratinocyte loss in the basal layer in response to fractionated radiotherapy was associated with growth arrest, terminal differentiation, and mitotic catastrophe that was sometimes followed by secondary apoptosis. During the first 4 weeks of daily treatment with 2 Gy only 0.5 apoptotic cells/mm was observed at most; a peak in apoptotic cells of 1.5 cells/mm was reached at the end of 5 weeks of treatment, including both melanocytes and keratinocytes in the basal layer. Thus, we can conclude that apoptosis among epidermal melanocytes was negligible. We assume that the protection of melanocytes from apoptosis was governed by BMI1, along with the MITF/Bcl-2 pathway.

**Figure 4:**
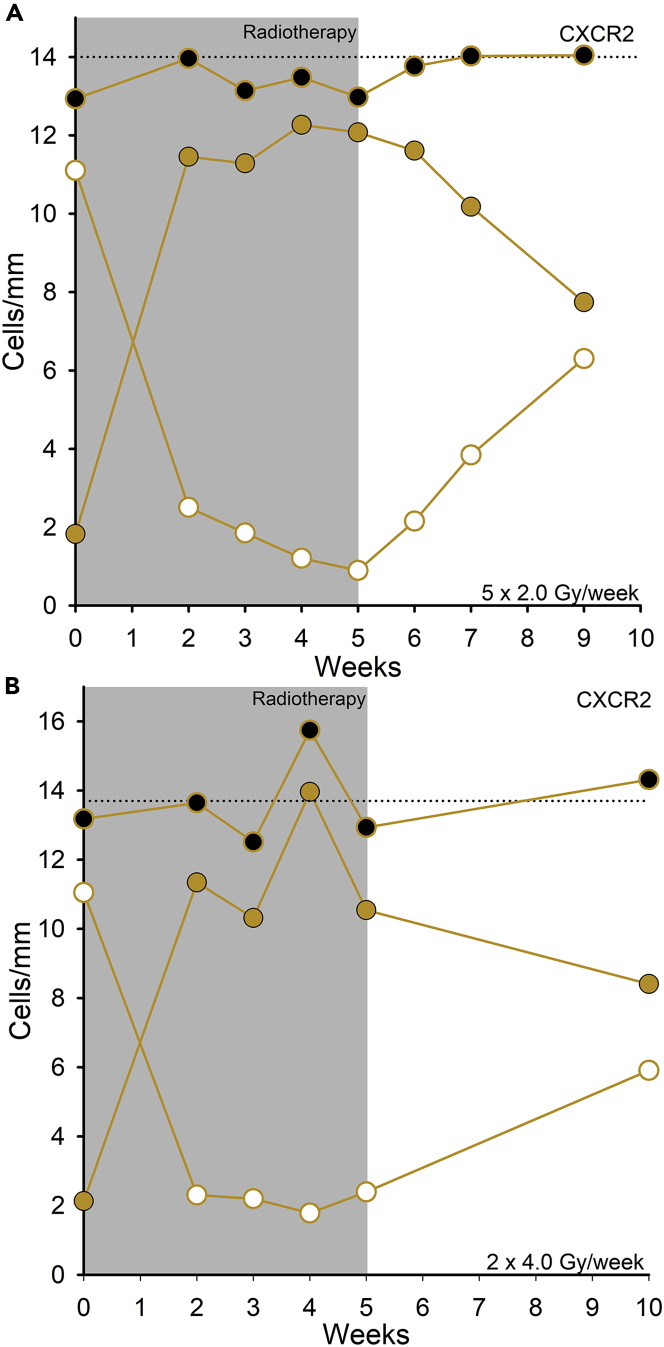
CXCR2 upregulation in melanocytes upon radiation exposure Upregulation of the cell membrane receptor CXCR2 for two patients receiving: (A) 5 × 2 Gy/week for 5 weeks and (B) 2 × 4 Gy/week for 5 weeks. CXCR2-positive melanocytes. () and CXCR2-negative cells () were morphologically characterized as melanocytes, and the total number of stained and unstained melanocytes is indicated (). Reference line represents the mean number of melanocytes counted in eosin-PAS staining (Turesson et al., 2020).

Melanocytes with accumulated DNA damage are expected to be lost mainly by senescence, with senescent cells eliminated by innate immunological cells. An early indication of senescence is the upregulation of the cell membrane receptor CXCR2 (Acosta et al., 2008; Kuilman et al., 2008). We assessed the expression of CXCR2 in two patients, each of whom received 5 times 2 Gy/week for 5 weeks and 2 times 4 Gy/week for 5 weeks, respectively; these patients were participants in a previous study (Turesson et al., 2020). CXCR2 expression at the melanocyte cell membrane was clearly visible at 1000×magnification. In unexposed skin, only a few cells stained positive for CXCR2. During the radiotherapy, the number of stained melanocytes increased, and by the end of 5 weeks of treatment, all melanocytes exhibited cell membrane staining, which started to decline post-treatment. Both patients expressed the same CXCR2 response (Figure 4). Of note, we observed CXCR2-stained melanocytes also in the hair follicle bulge. The keratinocytes were not stained, but endothelial cells and fibroblasts in the dermis showed upregulated CXCR2 receptors upon irradiation.

### Melanocyte proliferation

To determine if interfollicular melanocytes were proliferating, we stained cells for HTA28 for the detection of all stages of mitosis and Ki-67 for counting of cycling cells, and also the cell cycle proteins cyclin A and cyclin B1. In addition, we stained for pRb phosphorylation at serine 807/811, necessary for the cell to transit from G0 to G1 (Ren and Rollins, 2004). This staining enables the most accurate quantification of the proportion of proliferating melanocytes. In the immunostaining for HTA28, all cells in the melanocyte lineage in both unexposed and exposed skin were negative (i.e., no mitosis was observed). A total of 4 out of 1657 (0.2%) epidermal melanocytes were identified as positive for Ki-67. Double staining for ΔNp63 and Ki-67 did not reveal cycling melanocytes in unexposed skin or during treatment among 260 and 620 counted epidermal melanocytes, respectively (Figure S1D). In contrast, in the biopsies taken at 1, 2, and 3 weeks post-treatment, a total of 3 Ki-67-positive cells were identified out of 500 epidermal melanocytes.

**Figure 5:**
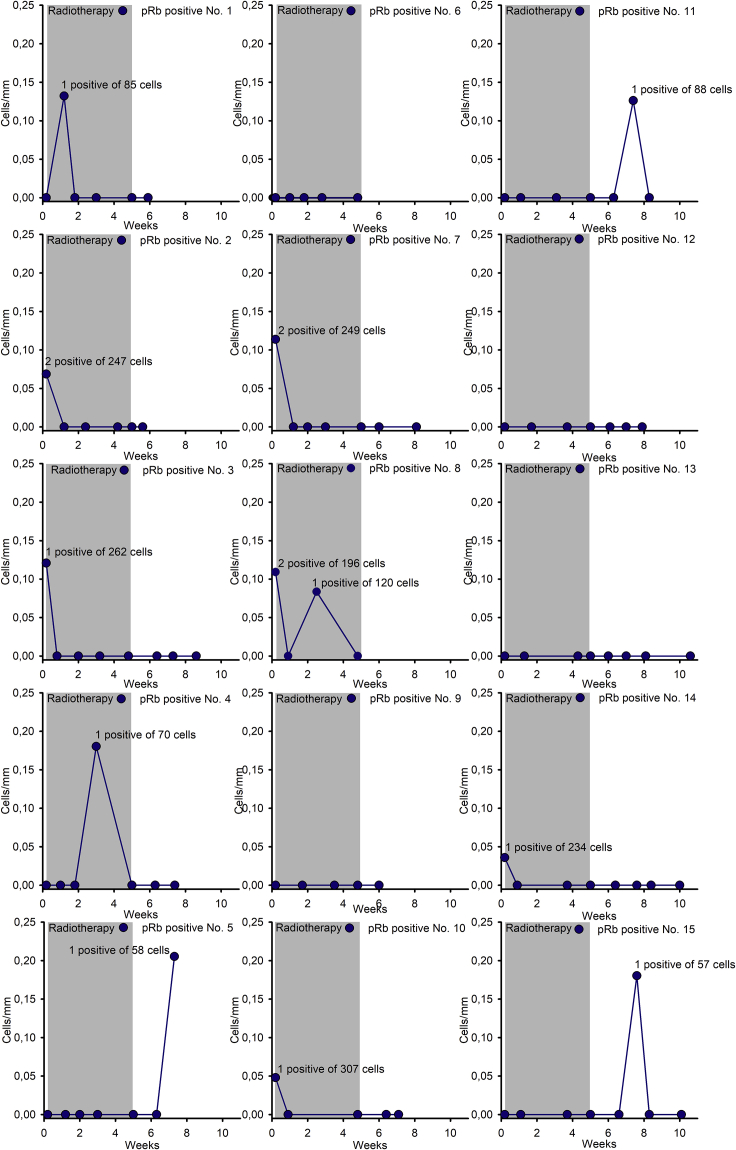
The number of interfollicular melanocytes expressing pRb in individual patients Number of stained cells per millimeter in the basal layer for 15 patients expressing pRb-positive cells ().

We immunostained biopsies from five patients for cyclins A and B1 to assess cell cycle progression of epidermal melanocytes through S, G2, and M phases and found 1 of 1019 melanocytes positive for cyclin A and none of 908 melanocytes positive for cyclin B1 in unexposed skin (Figures S8A and S8B). All melanocytes were negative for cyclins A and B1 during treatment. Post-treatment, 3 out of 1636 melanocytes were positive for cyclin A, and 2 out of 1545 melanocytes were positive for cyclin B1. We stained pRb for all 15 patients and observed it only in the nucleus (Figure S10). Only 15 positive cells, i.e. being within the cell cycle, were identified from a total of 10395 epidermal melanocytes from all 15 patients; the numbers of pRb-positive cells for each individual patient are presented (Figure 5). The stained cells were fairly evenly distributed over 10 weeks, confirming that melanocyte proliferation was minimal during the assessment period (Figure 5 and Table 1). The proliferation indices were as follows: in unexposed skin, 6 of 2223 melanocytes (0.3%); slightly lower during treatment (p = 0.10). Then 6 of 5839 melanocytes (0.1%) pRb-positive cells were observed, and remained at that level post-treatment, showing 3 positive melanocytes out of 2333 (0.1%). Thus, in the interfollicular epidermis, melanocyte renewal occurred but was minimal before and somewhat suppressed during and up to 5 weeks after the radiotherapy course. Of note, the occurrence of epidermal melanocytes expressing pRb as well as Ki-67, Cyclin A, and Cyclin B1 supports this statement. However, we cannot exclude the possibility that proliferation accelerated beyond that point in time.

Altogether, no migration from the hair bulge, negligible apoptosis, and minimal proliferation explains the undisturbed number of interfollicular melanocytes during the radiotherapy course and post-treatment. Epidermal melanocytes exhibit a treatment-induced increase in SOX10, MITF, and DCT expression that declined post-exposure, i.e. reversible differentiation.

## Discussion

### Induction of differentiation and dedifferentiation

With the clinical assay used in this study, we could show that a prolonged period of genotoxic damage induced reversible differentiation that was especially notable for a subset of undifferentiated melanocytes in the interfollicular epidermis. During the 5-week course of radiotherapy, this subgroup had an obvious temporary increase in SOX10, MITF, and DCT expression, which declined within 5 weeks after the end of treatment. SOX10-and MITF-negative cells in the unexposed skin almost disappeared after 5 weeks of radiotherapy, becoming SOX10-and MITF-positive cells, but within 5 weeks post-treatment, the SOX10-and MITF-negative cells re-established their original numbers. A similar pattern was observed for DCT. The temporary radiotherapy-induced increase in MITF activity was associated with simultaneous detection of Bcl-2 activity and its similar decline after the end of treatment. Using double staining and assessment in immunofluorescence, we previously showed a one-to-one correspondence between MITF and Bcl-2 (Fesse et al., 2019). The close relationship between MITF and Bcl-2 was confirmed in the present study. The strongest support for radiation-induced differentiation was the increasing number of DCT-expressing cells with more pronounced dendrites at the end of 5 weeks of treatment. The decline in DCT-positive cells during the subsequent weeks suggests that reversible differentiation occurred. Of note, this reasoning is based on the fact that the number of interfollicular melanocytes remained unchanged throughout the whole 10-week assessment period; the conclusion is justified by our findings that both apoptosis and proliferation were negligible and that the migration of immature melanocytes from the bulge of the hair follicles to interfollicular epidermis could be excluded in our assay.

Activation of the PAX3/SOX10/c-AMP complex is necessary for the transcription of MITF and its initiation of differentiation and melanin synthesis. The transcription factors PAX3 and SOX10 are both expressed in interfollicular melanocytes in adult skin and prevent terminal differentiation of this subset (Harris et al., 2013; Kubic et al., 2008; Lang et al., 2005). In unexposed skin, constitutive transforming growth factor (TGF)-β signaling from adjacent keratinocytes suppresses PAX3, maintaining the melanocytes in a quiescent non-proliferative state with a low level of MITF (Cui et al., 2007; Moustakas, 2008; Yang et al., 2008). Radiation exposure represses the paracrine TGF-β signaling from keratinocytes, consequently upregulating PAX3, which is most apparent in immature melanocytes (Fesse et al., 2019).

Repression of the TGF-β signal is mediated by p53 through the ATM/ATR/p53 and JNK pathways, which are activated by both UV and ionizing radiation (Lopez-Camarillo et al., 2012; Shiloh and Ziv, 2013; Yang et al., 2008), resulting in the phosphorylation and stabilization of the p53 protein in keratinocytes. Stabilized p53 protein exerts several paracrine regulatory functions on melanocytes. First, p53 suppresses TGF-β secretion from keratinocytes, resulting in the upregulation of PAX3 in melanocytes. Second, p53 induces the secretion of α-melanocyte-stimulating hormone (MSH), which activates the melanocortin-1 receptor (MC1R) on melanocytes. The α-MSH/MC1R complex induces c-AMP synthesis in melanocytes (Cui et al., 2007; Yang et al., 2008). c-AMP acts through the c-AMP response element-binding protein to enhance MITF expression. Under genotoxic stress, SOX10 is upregulated independently of p53 through the inhibition of ATR function in melanocytes (Ho et al., 2012). Thus, the upregulation of all three factors (PAX3, c-AMP, and SOX10) is triggered by UV and ionizing radiation, a prerequisite for the MITF transcription that pushes melanocytes toward differentiation. Stabilized p53 protein also induces stem cell factor (SCF) expression in keratinocytes. The paracrine secretion of SCF, the ligand of the c-KIT receptor on melanocytes (Murase et al., 2009), results in SCF/c-KIT signaling, which activates MITF (Grichnik et al., 1996; Hou and Pavan, 2008).

Previously, we identified a subpopulation of undifferentiated interfollicular melanocytes that do not express MITF or Bcl-2 (Fesse et al., 2019), and also melanocytes lacking both PAX3 and SOX10 expression, as well as c-KIT- and DCT-negative melanocytes. A dose-dependent upregulation of all of these markers occurred during treatment with daily radiotherapy doses in the range of 0.05-1.1 Gy delivered over 7 weeks. This was already observed after the lowest dose at 1-week treatment and confirmed at the treatment end, indicating that differentiation was the primary response to ionizing radiation. However, no post-treatment assessments were performed to reveal dedifferentiation.

Here, we confirmed the existence of a subpopulation of undifferentiated melanocytes that constitutes approximately 30% of all epidermal melanocytes. Daily radiotherapy doses of 2 Gy for 5 weeks induced differentiation and resistance to cell death and did not stimulate the melanocytes to proliferate. Termination of the treatment course resulted in dedifferentiation, and the undifferentiated melanocytes returned to their pre-radiation exposure numbers within 5 weeks post-treatment. We suggest that the most straightforward explanation for the dedifferentiation of the melanocytes was interrupted paracrine regulation by p53 at the termination of radiation exposure. The period of time required to reach the pre-treatment dedifferentiated state and restore the subset of immature interfollicular melanocytes may have been determined by the half-life of the degradation of the essential transcription factors and enzymes required for promoting differentiation.

In addition to its paracrine control by keratinocyte TGF-β, PAX3 in melanocytes is regulated by the E3 ligase APC/C (Cdh1) (Cao et al., 2015). APC/C (Cdh1) has a tumor suppressor function in melanocytes mediated by the subunit Cdh1, which inactivates PAX3 through ubiquitination and degradation, implying consequent inhibition of MITF production. UV irradiation triggers proteolysis of Cdh1, increasing the amount of stabilized PAX3 protein and facilitating differentiation. Once radiation exposure ends, PAX3 declines again through degradation by restored Cdh1, allowing cells to dedifferentiate.

The temporary differentiation of interfollicular melanocytes upon prolonged exposure to DNA damage in individuals undergoing a 5-week radiotherapy course is a unique finding. This phenomenon could be observed for each patient thanks to the individual dose-response created for each marker, minimizing the confounding factor of individual variability in the number of epidermal interfollicular melanocytes. We have identified a subset of immature interfollicular melanocytes that initiate and maintain differentiation during treatment with ionizing radiation. Melanocytes in the same subset can dedifferentiate post-treatment to the original number of immature melanocytes. We can exclude the migration of immature melanocytes from the bulge of the hair follicles (Goldstein et al., 2015) because the total administered dose of 50 Gy sterilizes hair follicles permanently (Lawenda et al., 2004). Of note, our clinical skin assay was based on biopsies taken from skin areas mostly unexposed to sun throughout life, both in the previous study on patients with prostate cancer (Fesse et al., 2019) and in the current study on patients with breast cancer, giving us an optimal opportunity to reveal the behavior of the actual number of less differentiated interfollicular melanocytes.

Apoptosis of the interfollicular melanocytes was negligible. Of note, cell division occurred among interfollicular melanocytes, although at a very low level, confirming an autonomous self-renewing melanocyte population in the epidermis. That self-renewal occurred among the epidermal melanocytes was proved by the demonstration of melanocytes expressing Ki-67, Cyclin A, Cyclin B1, and pRb at serine 807/811.

The current view of the melanocyte response to ionizing radiation is based on the study by Inomata et al. (2009) of hair follicles in adult mice. They found that after a single dose of ionizing radiation of 5 Gy, the melanocyte stem cells in the bulge region exhibited terminal differentiation and MC1R-mediated melanogenesis. As a result, the stem cell number declined because of inhibited self-renewal and irreversible hair graying was observed, and neither apoptosis nor senescence could be established. Dedifferentiation post-exposure was not reported to occur in that study. These earlier results and our current findings suggest a difference in response to DNA damage between the epidermal melanocyte-keratinocyte unit and the melanocyte stem cell-keratinocyte association in the niche within the bulge of the hair follicle. Indeed, our work adds to their results with our finding of absolute radioresistance of interfollicular melanocytes mediated through a temporary differentiation under delivery of 50 Gy, facilitated by adjacent keratinocytes, although substantially reduced in number at this radiation dose, (Turesson et al., 2020), while the same dosage regimen destroys the hair follicle. This suggests that interfollicular melanocytes, independent of the degree of differentiation, tolerate a much higher level of genotoxic stress than the melanocyte stem cells in the hair bulge. This distinction implies the existence of two independent cell-autonomous machineries for melanin synthesis and genotoxic response.

The presence of an autonomous interfollicular melanocyte population including immature and actively cycling melanocytes has thus far been demonstrated only in the hairless mouse tail (Glover et al., 2015). The currently accepted model in humans is based on studies of patients with vitiligo, assuming that the immature melanocytes present in the interfollicular epidermis of adults have migrated from the bulge of the hair follicles (Birlea et al., 2017).

Several mechanisms could explain the lack of nuclear p53 expression in the melanocytes upon exposure to radiotherapy. PAX3 can inactivate the p53 protein through ubiquitination and proteasomal degradation (Wang et al., 2011). The high BMI1 expression in melanocytes identified in this study may have attenuated the p53-mediated response to genotoxic damage, as BMI1 can bind directly to the p53 protein to ubiquitinate and degrade it (Calao et al., 2013). In addition, phosphorylation and subsequent stabilization of the p53 protein are prevented in melanocytes upon genotoxic damage through RhoJ inactivation of the ATR kinase (Ho et al., 2012).

The various pathways that suppress p53 expression in melanocytes also prevent p53-mediated transcription of p21. The increase in p21 mRNA and protein levels observed upon exposure to UV or ionizing radiation in melanocytes (Kulesz-Martin et al., 2005) is expected to be induced by MITF activation through its binding to the p21 promoter. This association has been demonstrated *in vitro*, suggesting that MITF regulates cell cycle progression through p21 (Carreira et al., 2005). In contrast, the *in situ* results presented here revealed a near total lack of nuclear p21 immunostaining in interfollicular melanocytes. The repression of p21 may be at least partly exerted by BMI1 via its binding to the p21 promoter (Subkhankulova et al., 2010). The lack of BMI1 expression *in vitro* (Basu et al., 2019) may explain the discrepancy in the upregulation of p21 in the scenario investigated by Carreira et al. (2005). Of note, only a small amount of p21 is necessary to prevent pRb phosphorylation and arrest cells in G0 (Overton et al., 2014; Spencer et al., 2013; Turesson et al., 2020; Yang et al., 2017). During radiotherapy in the present study, hardly any melanocytes were positive for any of the proliferation markers, suggesting that virtually all epidermal melanocytes were arrested in G0 during treatment.

Although nuclear p21 protein regulates cell cycle progression, cytoplasmic p21 inhibits apoptosis. The AKT and ERK kinases, when activated upon exposure to UV or ionizing radiation (Bonner et al., 1998; Gupta et al., 2001; Kadekaro et al., 2005; Lin and Fisher, 2007), both act to localize p21 to the cytoplasm. Cytoplasmic p21 protein binds to procaspase 3 and prevents its activation to caspase 3, blocking both the extrinsic and intrinsic apoptotic pathways (Cmielova and Rezacova, 2011; Heo et al., 2011; Sohn et al., 2006). Toward the end of treatment in this study, a significant number of interfollicular melanocytes exhibited cytoplasmic p21 staining, suggesting a real anti-apoptotic effect of p21.

Expression of the CXCR2 receptor on the cell membrane is an early sign of a premature senescent response, which is mediated by NF-kappa/β and C/EBPβ (Acosta et al., 2008; Gorgoulis et al., 2019; Ha et al., 2017; Kuilman et al., 2008). In the search for a senescence marker for dermal endothelial cells and fibroblasts, we discovered that CXCR2 was upregulated exclusively on interfollicular melanocytes in epidermis. When the treatment ended all epidermal melanocytes expressed CXCR2 in their cell membrane, and then declined post-treatment. However, the upregulation of CRXC2 was not associated with an obvious melanocyte reduction. Both UV and ionizing radiation activate NF-kappa/β, stimulating interleukin (IL)-6 and IL-8 secretion from melanocytes (Decean et al., 2013; Takabe et al., 2019). Both mediate their signals via extracellular binding to the CXCR2 receptor. Surprisingly, the CXCR2 expression also was transient, and the number of melanocytes expressing CXCR2 declined successively after the treatment end (Figure 4). The temporary CXCR2 expression of the melanocytes reinforces protection against apoptosis during genotoxic exposure (Childs et al., 2014) and prevents the development of an irreversible senescent phenotype post-treatment.

Ultimately, the senescent state is regulated by Notch1. Senescence instigation by the DNA-damage response is regulated by transient induction of Notch1 signaling. Notch1 activation suppresses C/EBPβ, which results in reduced IL-6 and IL-8 secretion, preventing the recruitment of innate immune cells and elimination of CXCR2-expressing melanocytes in a pre-senescent state (Hoare et al., 2016). This pattern is in line with our observation that the number of melanocytes remained unchanged for a long irradiation period despite the upregulation of CXCR2. We have confirmed that CXCR2 is upregulated already after daily doses of 0.05 Gy (unpublished). However, it remains to be proven that the DNA damage induced by UV radiation is enough to upregulate CXCR2.

In a search to identify keratinocyte stem cells in the basal layer of the epidermis, we performed BMI1 staining and found that only the melanocytes expressed detectable amounts of this protein. In unexposed skin, all but a few interfollicular melanocytes presented nuclear BMI1, and the expression became even more pronounced during the irradiation period, with only 0.4 BMI1-negative cells/mm left at the end of treatment. Based on the current literature, we surmise that the most prominent tasks of BMI1 in melanocytes following DNA insults are to prevent apoptosis and terminal differentiation by suppressing the expression of p53 and p21 proteins, and also to inhibit permanent cell-cycle arrest through senescence by suppressing the expression of p16 protein. In this way, BMI1 will maintain the self-renewal capability of interfollicular melanocytes, despite long-term genotoxic exposure as with the 5 weeks of radiotherapy in our study.

How is BMI1 regulated in melanocytes? Notch1 induces the transcription of BMI1 in a cellular context-dependent manner (Katoh and Katoh, 2020). In the small intestine, the constitutive transcription of BMI1 in normal stem cells and their undifferentiated progenitor cells results from the interplay of activated Notch1 and β-catenin, and BMI1 is involved in the self-renewal of the stem cells (Lopez-Arribillaga et al., 2015). Epidermal melanocytes in culture do not express BMI1 protein (Basu et al., 2019). Therefore, the high expression of BMI1 in melanocytes is most likely regulated by paracrine signaling from neighboring keratinocytes, and the physiological activity of the Notch1 and Wnt/β-catenin pathways may be responsible for BMI1 transcription and constitutive expression of BMI1 protein (Bellei et al., 2011; Seleit et al., 2014), as demonstrated in the stem cells in the small intestine. The p53 protein induces Notch1 activity by binding to its promoter in keratinocytes (Lefort et al., 2007) and is thought to enhance Notch1 signaling in melanocytes upon genotoxic exposure (Moriyama et al., 2006). Notch1 co-operates with β-catenin stabilized by α-MSH and Wnt signaling from keratinocytes (Bellei et al., 2011; Yamada et al., 2013). Whether these mechanisms are responsible for increased BMI1 expression in interfollicular melanocytes upon irradiation remains to be confirmed. Of interest, a previous study revealed that β-catenin immortalizes melanocytes by suppressing p16/INK4A expression (Delmas et al., 2007). Our current findings suggest that the mechanism can be ascribed to the facilitation of BMI1 by β-catenin.

**Figure 6:**
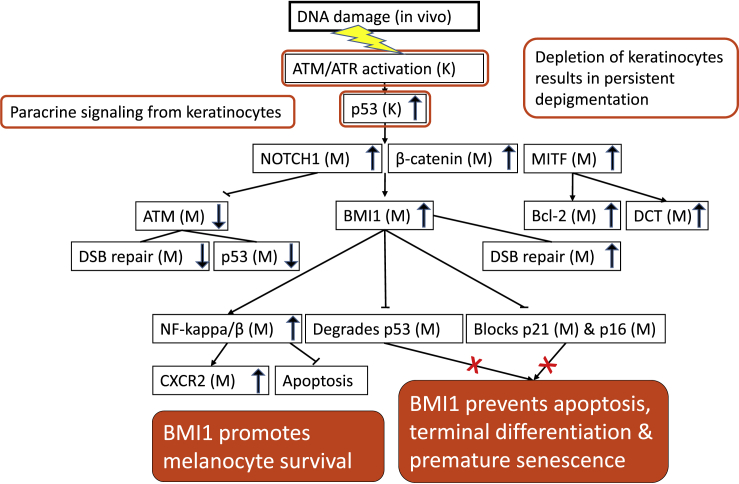
Flow chart of possible molecular pathways regulating epidermal melanocyte survival *in situ* upon genotoxic exposure M: melanocyte K: keratinocyte

**Figure 7:**
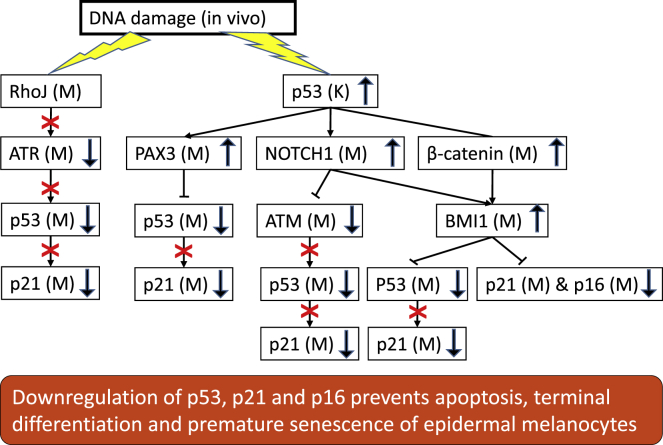
Flow chart of possible molecular pathways regulating p53-p21 expression in interfollicular melanocytes *in situ* upon genotoxic exposure M: melanocyte K: keratinocyte

The potential mechanisms discussed above are summarized in flow charts showing possible molecular pathways regulating epidermal melanocyte survival *in situ* and p53/p21 protein as well as p16 protein expression in interfollicular melanocytes upon genotoxic exposure (Figures 6 and 7).

### Melanocyte apoptosis and cell cycle progression

Upon moderate exposure of normal skin to UV radiation, epidermal melanocytes are resistant to apoptosis (Bowen et al., 2003; Hornyak et al., 2009), which is ascribed to the upregulation of the anti-apoptotic protein Bcl-2 by MITF (McGill et al., 2002). Resistance to ionizing radiation also was manifested in the current study and associated with a strong correlation between MITF and Bcl-2 expression in our patient cohort, confirming the one-to-one correspondence between these two markers revealed previously by double staining (Fesse et al., 2019). A maximum of approximately 1.5 apoptotic cells/mm among basal cells including both keratinocytes and melanocytes was reached at the end of 5 weeks of treatment in the current patient cohort, and a successive decrease in apoptotic cell numbers was noted post-treatment (Turesson et al., 2020). Therefore, we concluded that apoptosis was not a common event in the melanocyte response to ionizing radiation. Consequently, from the numerical findings, the reduction in SOX10-, MITF-, and Bcl-2-negative cells from about 5 to 1 per mm during 5 weeks of radiotherapy could not be explained by apoptosis.

The scarcity of interfollicular melanocytes makes it difficult to establish their proliferation rate. The very slow self-renewal among epidermal melanocytes identified in the assessments of HTA28, Ki-67, cyclin A, and cyclin B1 was confirmed by staining for pRb phosphorylated at serine 807/811. Stained cells were observed very sporadically before (0.3%), during (0.1%), and following radiotherapy (0.1%). Although proliferation occurred during the recovery phase after radiation-induced DNA damage, the very low figures could not explain the increase in the number of SOX10-, MITF-, and Bcl-2-negative cells after the completion of the radiotherapy course.

In contrast to our findings, Yamaguchi et al. (2008) reported a 2- to 3-fold increase in epidermal melanocyte density after repetitive UV exposure, which others confirmed (Brenner et al., 2009; Coelho et al., 2009). In the latter studies, no proliferation was observed with the use of cyclins D and E or PCNA as cell cycle markers. The lack of proven proliferation upon UV exposure was interpreted as melanocytes migrated from the hair follicle bulges to interfollicular epidermis, accounting for the pronounced increase in melanocyte density upon repeated UV irradiation (Goldstein et al., 2015). By comprehensively quantifying different markers in thousands of epidermal melanocytes, we established that subcutaneous interfollicular melanocytes are an autonomous cell population with slow cell renewal and different degrees of differentiation. Furthermore, we found that the number of cells in the melanocyte lineage in the interfollicular epidermis remained undisturbed during the course of radiotherapy and several weeks post-treatment. The proportions of undifferentiated and differentiated melanocytes at the beginning of radiotherapy shifted toward more differentiated cells, and the majority of epidermal melanocytes were differentiated by the end of treatment. Of importance, the proportions of undifferentiated and differentiated melanocytes returned to their pre-treatment levels within 5 weeks after the therapy ended. We propose that the high BMI1 expression in interfollicular melanocytes, under paracrine control of keratinocytes, could be primarily responsible for the management of DNA damage, through suppression of the nuclear accumulation of p53, p21, and p16 proteins. Furthermore, BMI1 can inhibit apoptosis by the up-regulation of NF-kappa/β-CXCR2 signaling during genotoxic stress. The CXCR2 declined post-exposure, indicating also temporary premature senescence activity, preventing proliferation.

If these mechanisms can be confirmed, adjacent keratinocytes protect interfollicular melanocytes from death by conferring stem cell properties on them, leading to an assurance of melanin availability for the protection of themselves against UV radiation. The DNA-damage response of melanocytes is largely regulated by the DNA-damage response of keratinocytes and mediated primarily by stabilized p53 protein induced to a similar degree by UV and ionizing radiation. Thus, we can assume that our findings are also true for UV radiation. The less-differentiated subset of interfollicular melanocytes is more vulnerable to DNA damage than more differentiated melanocytes (Yamaguchi et al., 2006). According to our findings, intermittent radiation exposures at intervals of 5 weeks or longer will re-sensitize the immature melanocytes to each exposure event. The establishment of very slow cell renewal and prolonged longevity conferred on epidermal interfollicular melanocytes by their specific response to DNA damage have implications for melanomagenesis. Differentiation induced by radiation exposure over many weeks followed by dedifferentiation allows epidermal melanocytes to survive and potentially experience many UV exposure events. In this way, interfollicular melanocytes can accumulate unrepaired DNA damage to a great extent over time, which may cause the malignant transformation to melanoma in the long run. The immature interfollicular melanocytes have the greatest risk to suffer permanent changes in DNA. Recent findings offer support for increased vulnerability to genetic damage for the immature subset of interfollicular melanocytes to intermittent UV radiation exposure compared with continuous (Tang et al., 2020). We emphasize that our findings and interpretations of the fate of interfollicular melanocytes upon genotoxic damage are only hypothesis-generating for future studies searching for the mechanisms in-depth.

There are other clinical aspects of this study. Work from decades ago used reflectance spectrophotometry to determine the degree of pigmentation induced after different fractionation schedules and dose levels up to at least 6 months post-radiotherapy (Turesson and Notter, 1984). At the end of the assessment, a weak persistent pigmentation was observed compared with unexposed skin, not always obvious to the unaided eye but with individual variability in degree. We propose that persistent unrepaired DNA damage maintains long-lasting differentiation and melanin synthesis. Because of the paracrine dependency of keratinocytes for melanin synthesis and survival, melanocytes will die as a consequence of the depletion of keratinocytes, resulting in permanent depigmentation. This is a well-known phenomenon commonly seen after acute moist skin reactions induced by radiotherapy doses above 50 Gy through almost complete depletion of epidermal keratinocytes.

### Limitations of the study

The findings based on immunostaining in the study suggest that the keratinocytes adjacent to the interfollicular melanocytes provide them with stem cell properties and resistance to genotoxic damage. The main limitation of the study is that we have not identified the molecular pathways in the keratinocytes that exert the paracrine regulatory functions on the melanocytes, as the constitutive expression of BMI1. Another limitation is that we have not developed a technique for *in vivo* fate tracing of epidermal melanocytes to confirm the temporary differentiation under genotoxic stress and resistance to cell death.

## STAR★Methods

### Key resources table

**Table:** 

REAGENT or RESOURCE	SOURCE	IDENTIFIER
**Antibodies**		

mouse monoclonal p63 (1:200, ICH & IF)	Santa Cruz Biotechnology	Cat# sc-8431; RRID:AB_628091
rat monoclonal HTA28 (1:200, ICH)	Abcam	Cat# ab10543; RRID:AB_2295065
mouse monoclonal MITF (1:50, ICH)	Agilent Dako	Cat# M3621; RRID:AB_2142100
mouse monoclonal Bcl-2 (1:15 ICH, 1:20 IF)	Agilent Dako	Cat# M0887; RRID:AB_2064429
mouse monoclonal Ki-67 (1:100, ICH & IF)	Agilent Dako	Cat# M7240; RRID:AB_2142367
mouse monoclonal p53 (1:50, ICH)	Agilent Dako	Cat# M7001; RRID:AB_2206626
rabbit phosphorylated Rb (1:20, ICH)	Cell Signaling Technology	Cat# 8516; RRID:AB_11178658
mouse monoclonal p21 (1:100, ICH)	Millipore (Calbiochem)	Cat# OP64-20UG; RRID:AB_10683397
mouse monoclonal cyclin A (1:300, ICH)	Leica Biosytems (Novocastra)	Cat# NCL-CYCLIN A; RRID:AB_563675
mouse monoclonal cyclin B1 (1:50, ICH)	Leica Biosytems (Novocastra)	Cat# NCL-CYCLIN B1; RRID:AB_563676
mouse monoclonal BMI1 (1:100, ICH)	Millipore	Cat# 05-637; RRID:AB_309865
goat polyclonal SOX10 (1:100, ICH)	Santa Cruz Biotechnology	Cat# sc-17343; RRID:AB_2255319
mouse monoclonal CXCR2 (1:200, ICH)	BD Biosciences	Cat# 555932; RRID:AB_396229
rabbit polyclonal MITF (1:50, IF)	Atlas Antibodies	Cat# HPA003259; RRID:AB_1079381
mouse monoclonal p16 (1:10, ICH)	Novocastra	Cat# PA0016
rabbit polyclonal p63 (1:100, IF)	Atlas Antibodies	Cat# HPA058154; RRID:AB_2683624
mouse monoclonal DCT (1:500, IF)	Santa Cruz Biotechnology	Cat# sc-74439; RRID:AB_1130818
goat anti mouse Alexa Fluor 480 (1:100, IF)	Molecular Probes	N/A
goat anti rabbit Alexa Fluor 480 (1:100, IF)	Molecular Probes	N/A
goat anti mouse Alexa Fluor 555 (1:100, IF)	Molecular Probes (Thermo Fisher)	Cat# A-21424; RRID:AB_141780
goat anti rabbit Alexa Fluor 555 (1:100, IF)	Molecular Probes (Thermo Fisher)	Cat# A-21428; RRID:AB_141784

**Biological samples**		

Skin biopsies	15 breast cancer patients	N/A

**Chemicals, peptides, and recombinant proteins**		

DAPI di-lactate	Molecular Probes (Thermo Fisher)	Cat# D3571
Hematoxylin	Vector Laboratories	Cat# H-3401
Vectashield mounting medium	Vector Laboratories	Cat# H-1200-10
Citrate buffer pH 6.0	Thermo-scientific, Fremont, CA	N/A
Counterstainer	Meyers HTX	N/A
goat probes and goat polymers	Biocare Medical	Cat#GHP516

**Software and algorithms**		

Statistical Package for the Social Sciences	version 25, Chicago, IL, USA	N/A
SigmaPlot	version 12.5, SYSTAT software	N/A

**Other**		

Eclipse, 50i	Nikon	N/A
de-cloaking chamber	Biocare Medical, Walnut Creek, CA	N/A
Superfrost Plus slides	Menzel-Gläser, Germany	N/A
Ventana Benchmark automated stainer	Ventana Medical Systems, Tucson, AZ	N/A
Ventana View DAB detection kit	Ventana Medical Systems, Tucson, AZ	N/A

### Resource availability

#### Lead contact

Further information and requests for resources and reagents should be directed to and will be fulfilled by the Lead Contact and is listed in the Author List, Ingela Turesson (ingela.turesson@gmail.com).

#### Materials availability

All unique reagents generated in this study are available from the lead contact.

### Experimental model and subject details

#### Human subjects

Skin biopsies were taken from 15 breast cancer patients (median age 57 years; range 48–75 years) undergoing post-mastectomy radiotherapy with curative intent in Gothenburg, Sweden, from 2005 to 2007. The patients had lived their whole lives in Sweden and had skin type II-III according to Fitzpatrick (Fitzpatrick, 1988; Roberts, 2009). Approval for the study was obtained from the Ethics Committee at the University of Gothenburg. Written informed consent was received from all patients prior to participation.

### Method details

#### Radiotherapy

Radiotherapy was given to the thoracic wall with opposed tangential fields and photons of 5 MV. The prescribed dose was 25 fractions of 2.0 Gy daily for 5 weeks. A bolus of 5 mm in a 5- to 10-cm broad strip covered the surgical scar. At a depth of 0.1 mm below the skin surface, the dose per fraction relevant for the biological endpoints was determined to be very close to 2 Gy under the bolus.

#### Biopsies

Punch biopsies 3 mm in diameter were taken using local anesthesia with lidocaine hydrochloride (5 or 10 mg/mL) without adrenaline. All biopsies were fixed in 4% formaldehyde immediately after collection and coded, at the longest for 3 days before being embedded. Two unexposed control biopsies were taken prior to the computed tomography examination for planning the radiotherapy dose to avoid the radiation exposure caused at this occasion. During the treatment period, biopsies were taken from areas under the bolus to ascertain that a dose of 2 Gy per fraction was delivered to the basal layer of the epidermis. Multiple biopsies were taken from each patient at predetermined intervals before, during, and after treatment, with the latest taken at up to 5 weeks.

#### Immunohistochemistry

Three transverse tissue sections were taken from various levels of each biopsy and mounted on slides. The slides were dried overnight at 37°C. Immunohistochemical staining was performed on a Ventana Benchmark automated stainer using the Ventana View DAB detection kit and subsequent manual counterstaining. Immunohistochemistry for SOX10 was performed as described previously using a manual protocol (Lang et al., 2005). Briefly, following antigen retrieval using citrate buffer pH 6.0 in the decloaking chamber for 4 min at 125°C, the slides were cooled in the chamber, for a total time of 40 min. A goat polyclonal primary antibody for SOX10 was applied and incubated for 1 h in a humidity chamber. For detection, goat probes and goat polymers were used, and the slides were counterstained with hematoxylin.

#### Immunofluorescence

Combinations of primary antibodies from different species were used in the double staining to assess immunofluorescence. Double staining was detected using appropriate pairs of fluorescent secondary antibodies raised in goat or donkey and attached to the fluorescent dyes. The manual staining protocol included epitope retrieval in boric acid buffer (pH 7.0) heated in a water bath (90°C) for 45 min. Antibody incubations were performed at 20°C for 1 h, followed by three 5-min washes in PBS (pH 7.4). DAPI dilactate was used for nuclear staining. Slides with air-dried sections were mounted in the mounting medium.

For all immunostainings performed, tissues known to express the antigen of interest were used as positive controls. As negative controls, skin biopsy sections omitting the primary antibodies from the staining procedure were used. Of note, for each molecular marker, all tissue sections from one patient were stained simultaneously to avoid influence from fluctuations in the procedure.

### Quantification and statistical analysis

The total number of melanocytes with positive or negative immunostaining was counted per millimeter of the basement membrane on three separate sections for all biopsies. All counting was performed by one of the authors (PF) using a bright-field microscope at high power (1000× magnification). The number of melanocytes positive and negative for each marker, the number of biopsies and patients at each time point, from control biopsies up to 10 weeks are presented in Table 1. The main question is whether the number of melanocytes changes with RT and if the number then returns after the end of the treatment for each staining. Therefore, we divided the analysis into two steps - “during treatment” and “after treatment.” Each individual’s last follow-up opportunity for the treatment itself (approximately at the end of week 5) is thus included in both sub-analyses, first as an endpoint and then as a starting point. We used the total dose as an independent variable for the analysis of changes during the treatment period, and the number of days for the analysis of changes during the period after treatment. Due to the correlation of the data in the individual patient, a Linear Mixed Model with one independent variable in the analysis (“Total dose” or “Time”) was chosen, where the Linear Mixed Model analyzed dose-response relationship during treatment and time-response relationship after treatment. Complementary evaluation was performed with the Linear Mixed Model to test any change from the baseline value to that determined at a certain dose point during treatment or time point post-treatment; this approach reinforced the first analysis. p-values for increasing or decreasing trends were presented for each marker. Statistical Package for the Social Sciences (SPSS, version 25, Chicago, IL, USA) was used for all statistical analyses. All graphs were performed in SigmaPlot. Data are shown as means ± SEM unless otherwise specified. A p < 0.05 is considered statistically significant.

To evaluate the co-expression of two proteins in the double staining, subpopulations with positive or negative staining for each protein were identified. The proportion of co-stained cells was then estimated, and changes between unexposed, end of treatment and post-treatment were tested for significance using Fischer's exact test.

#### Comments on melanocyte density and radiation-induced differentiation revealed by immunostaining of molecular markers

ΔNp63-negative cells belonged to the melanocyte lineage. Small amounts of ΔNp63 in melanocytes cannot be excluded with the staining protocol used. As discussed previously (Fesse et al., 2019), this may be a confounding factor when using this marker, leading to a possible underestimation of the number of melanocytes in the order of magnitude 10%. Of note, ΔNp63 protein isoforms in melanocytes are not induced by ionizing radiation or UV radiation (Kulesz-Martin et al., 2005).

The number of ΔNp63-negative cells in a previous study of 33 prostate cancer patients was 17.4 cells/mm, with a coefficient of variation (CV) of 22% in unexposed skin (Fesse et al., 2019) compared to 18.1 cells/mm and a CV of 25% in the present study with 15 breast cancer patients. In both studies the average number of ΔNp63-negative cells over the assessment period was 18 cells/mm. Thus, no difference was seen between males and females. In addition, neither study showed a change in the number of ΔNp63-negative cells during 7 weeks of radiotherapy with daily dose fractions in the range of 0.05–1.1 Gy, and for daily 2.0- Gy fractions during 5 weeks and up to 5 weeks post-treatment. This was also the case for hypofractionation with 2.4 and 4.0 Gy per fraction, as well as for accelerated fractionation with 2 × 2.0 Gy/day assessed in eosin-PAS staining, using the same clinical model (Turesson et al., 2020).

The number of MITF-positive cells determined previously for prostate cancer patients was 12.7 ± 0.7 cells/mm in unexposed skin and 21.6 ± 1.7 cells/mm after 6.5 weeks of radiotherapy with 1.1 Gy per fraction (Fesse et al., 2019), compared with 14.1 ± 1.5 cells/mm in unexposed skin and 20.8 ± 1.5 cells/mm with 2.0 Gy per fraction after 5 weeks of radiotherapy in the present study. The melanocytes also were easily identified by Bcl-2 staining. The number of Bcl-2–positive cells determined in the prostate study was 14.7 ± 0.6 cells/mm in unexposed skin and 22.5 ± 1.0 cells/mm after 6.5 weeks of radiotherapy with 1.1 Gy per fraction, compared with 16.9 ± 1.2 Bcl-2–positive cells/mm in unexposed skin and 23.3 ± 1.1 cells/mm with 2.0 Gy per fraction after 5 weeks of radiotherapy in the present study.

In the current work, we identified the number of MITF-negative melanocytes and Bcl-2–negative melanocytes in the unexposed skin, 5.2 ± 0.3 cells/mm and 4.0 ± 0.4 cells/mm, respectively, compared with 4.9 ± 0.3 cells/mm and 4.6 ± 0.1 cells/mm, respectively, in the prostate study (Fesse et al., 2019). A subset of SOX10-negative cells was also identified in the prostate study (Fesse et al., 2019), and confirmed in the current study. The numbers of MITF-, Bcl-2–, and SOX10-negative cells decreased significantly during radiotherapy in both studies. At the completion of radiotherapy, each of these negative cell populations was reduced to approximately 1 cell/mm. This reduction in SOX10-, MITF-, and Bcl-2–negative melanocytes was reflected in a parallel significant increase in SOX10-, MITF-, and Bcl-2–positive melanocytes that indicated differentiation upon radiotherapy. The current study showed that these subsets of melanocytes with negative staining were restored to the original numbers within 5 weeks post-treatment, indicating that dedifferentiation occurred, as we could exclude migration from the hair bulge and had determined that proliferation was negligible.

#### Consistency in quantification of melanocyte markers in the present study

The total numbers per millimeter of positive and negative melanocytes determined for each marker over the 10 weeks of assessments displayed a high consistency in the quantification of the various immunohistochemical staining performed at 1000× magnification (Table S1). There was a good agreement between the markers except for slightly higher numbers for the Bcl-2 staining. Table S1 also presents the total number of interfollicular melanocytes counted at each time point for each staining.

Of note, the sensitivity of detection and the accuracy of the assessments of double staining in immunofluorescence were limited by the use of 400× magnification, and assessments performed on photos influenced by the brightness when taken. Therefore, these estimates should be regarded as semi-quantitative. The less accuracy and sensitivity were reflected in the larger proportions of negative cells we observed for all markers in the double staining and immunofluorescence compared with counting in classical immunohistochemically stained tissue sections.

However, the double staining and detection by immunofluorescence was essential to identify melanocytes co-expressing or not the specific markers of interest. The double staining was also important to confirm the pattern of the response of each marker (i.e. the relative changes with dose and time for an individual patient) determined more accurately by immunohistochemistry (Figures S2 and S6).

## Data Availability

• All other data reported in this paper will be shared by the lead contact upon request. • This paper does not report original code. • Any additional information required to reanalyze the data reported in this paper is available from the lead contact upon request.
