# Supplementary material for: Human cutaneous interfollicular melanocytes differentiate temporarily under genotoxic stress
Source: iScience. 2022 Sep 28;25(10):105238. doi: 10.1016/j.isci.2022.105238 (PMC9579029; doi:10.1016/j.isci.2022.105238)
Supplement: Document S1. Figures S1–S10 and Table S1 [file mmc1.pdf]

iScience, Volume 25

Supplemental information

Human cutaneous interfollicular melanocytes differentiate temporarily under genotoxic stress

Per Fessé, Jan Nyman, Ingegerd Hermansson, Maj-Lis Book, Johan Ahlgren, and Ingela Turesson

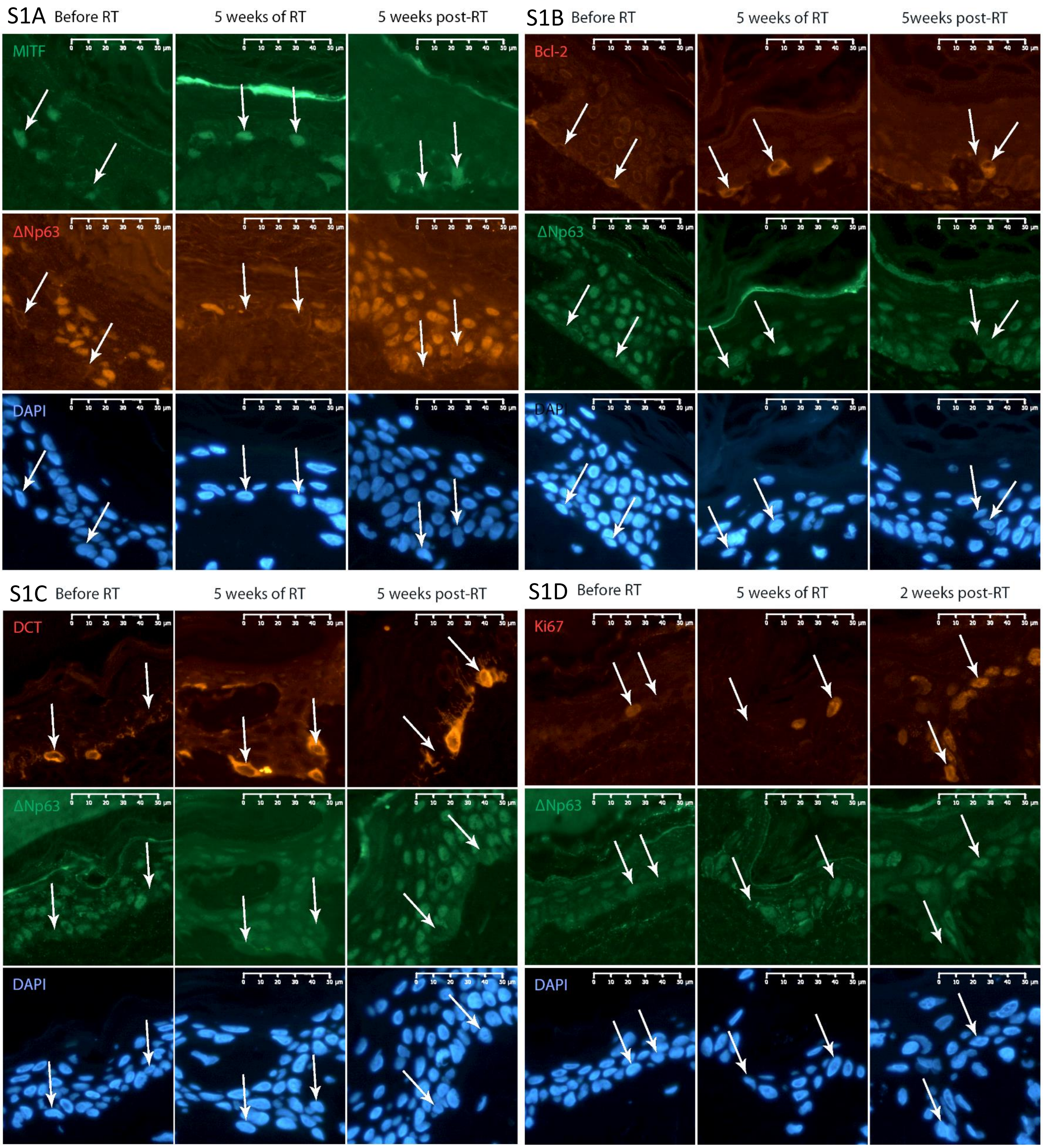
S1A
Before RT
5 weeks of RT
5 weeks post-RT
MITF
ΔNp63
DAPI
S1B
Before RT
5 weeks of RT
5weeks post-RT
Bcl-2
ΔNp63
DAPI
S1C
Before RT
5 weeks of RT
5 weeks post-RT
DCT
ΔNp63
DAPI
S1D
Before RT
5 weeks of RT
2 weeks post-RT
Ki67
ΔNp63
DAPI

Figure S1

Figure S1. ΔNp63-negative cells belong to the melanocyte lineage, and express various molecular markers in interfollicular melanocytes (related to main Figure 1 and 2)

Double-staining experiments of the epidermis illustrating the response of melanocytes to fractionated radiotherapy (RT). Staining patterns in biopsies acquired before treatment are compared to staining at time points during and after treatment. Cells in the melanocyte lineage are negative for ΔNp63; this staining visualizes the melanocyte population throughout RT. (S1A) Double staining for MITF and ΔNp63. Arrows in the left column respectively indicate a MITF-positive and a MITF-negative melanocyte in the basal layer prior to ionizing radiation (IR) exposure. Two MITF-positive melanocytes are highlighted by arrows in the middle column, illustrating the staining pattern after 5 weeks of IR exposure. Arrows in the right column indicate MITF-negative and MITF-positive melanocytes in the basal layer 5 weeks post-treatment. (S1B) Double staining for Bcl-2 and ΔNp63. Arrows in the left column respectively point to a Bcl-2–negative and a Bcl-2–positive melanocyte in the basal layer prior to IR exposure. Two Bcl-2–positive melanocytes are highlighted by arrows in the middle column, illustrating the staining pattern after 5 weeks of IR exposure. Arrows in the right column respectively indicate a Bcl-2–negative and Bcl-2–positive melanocyte in the basal layer 5 weeks post-treatment. (S1C) Double staining for DCT and ΔNp63. Arrows in the left column point respectively to a DCT-positive and DCT-negative melanocyte in the basal layer prior to IR exposure. Two DCT-positive melanocytes are highlighted by arrows in the middle column, illustrating the staining pattern after 5 weeks of IR exposure. Arrows in the right column respectively indicate a DCT-negative and a DCT-positive melanocyte in the basal layer 5 weeks post-treatment. (S1D) Double staining for Ki-67 and ΔNp63. Arrows in the left column respectively indicate a Ki-67–positive keratinocyte and a Ki-67–negative melanocyte in the basal layer prior to IR exposure. A Ki-67–negative melanocyte and a Ki-67–positive keratinocyte each are highlighted by arrows in the middle column, illustrating the staining pattern after 5 weeks of IR exposure. Arrows in the right column respectively indicate a Ki-67–positive melanocyte and a Ki-67–positive keratinocyte in the basal layer 2 weeks post-treatment.

S2

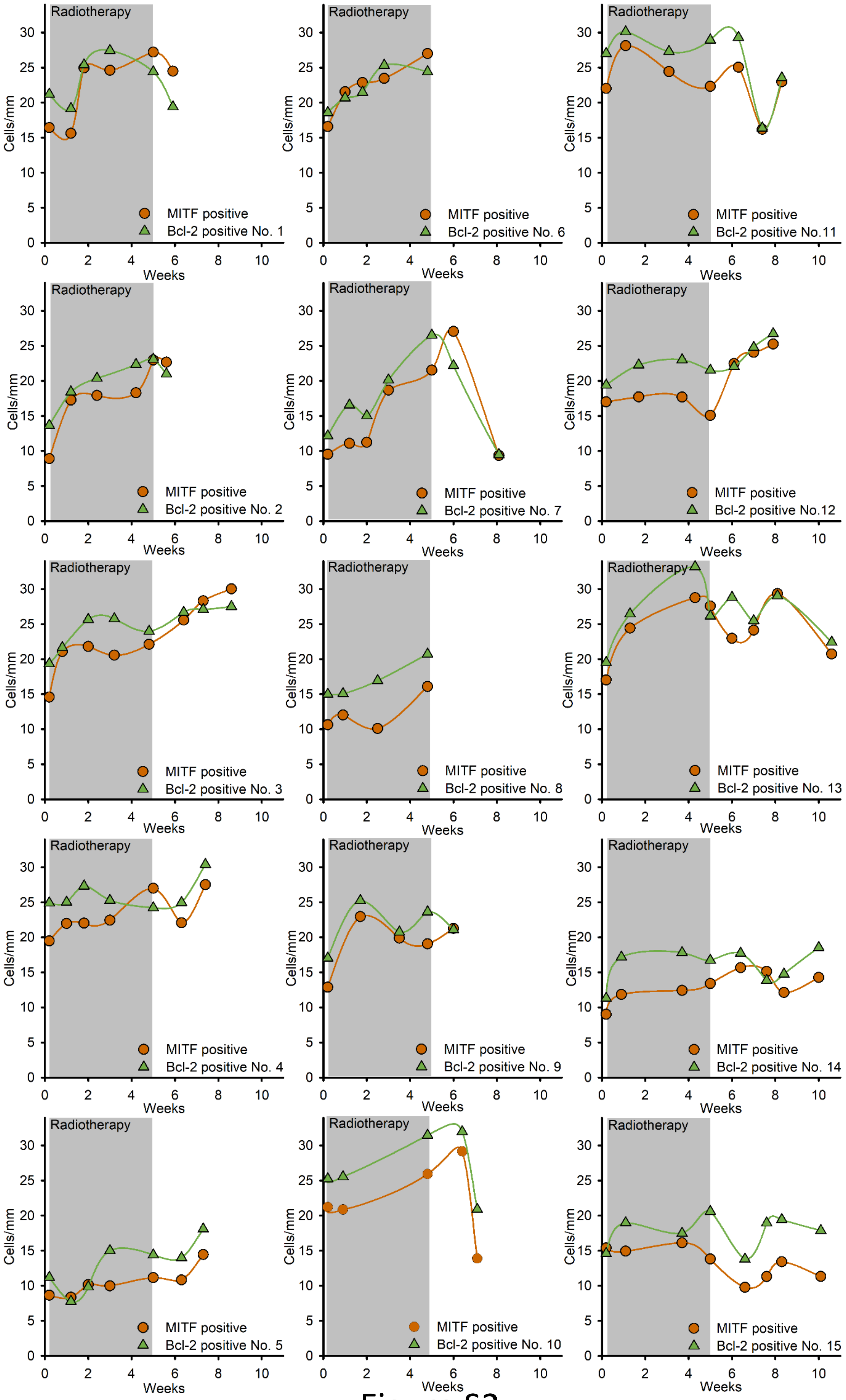
Radiotherapy
Cells/mm
Weeks
MITF positive
Bcl-2 positive No. 1
Bcl-2 positive No. 6
Bcl-2 positive No.11
Bcl-2 positive No. 2
Bcl-2 positive No. 7
Bcl-2 positive No.12
Bcl-2 positive No. 3
Bcl-2 positive No. 8
Bcl-2 positive No. 13
Bcl-2 positive No. 4
Bcl-2 positive No. 9
Bcl-2 positive No. 14
Bcl-2 positive No. 5
Bcl-2 positive No. 10
Bcl-2 positive No. 15
0
5
10
15
20
25
30
0
2
4
6
8
10

Figure S2

Figure S2. The number of interfollicular melanocytes expressing MITF and Bcl-2
(related to main Figure 2 and Table 1)

Number of cells per millimeter in the basal layer for each patient (n = 15 patients) expressing MITF-positive melanocytes (●) and Bcl-2–positive melanocytes (▲).

S3

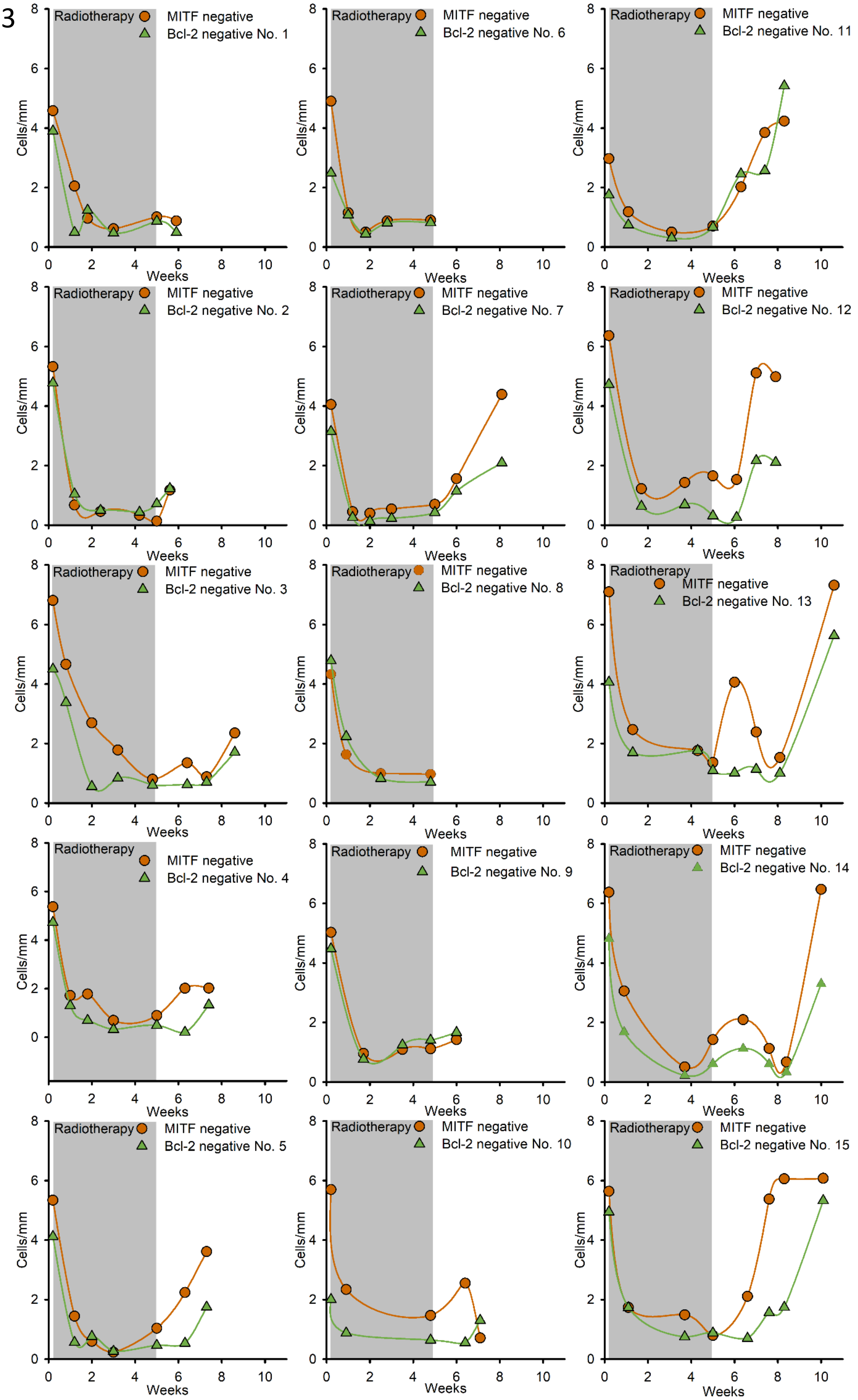

Radiotherapy
MITF negative
Bcl-2 negative No. 1
Bcl-2 negative No. 2
Bcl-2 negative No. 3
Bcl-2 negative No. 4
Bcl-2 negative No. 5
Bcl-2 negative No. 6
Bcl-2 negative No. 7
Bcl-2 negative No. 8
Bcl-2 negative No. 9
Bcl-2 negative No. 10
Bcl-2 negative No. 11
Bcl-2 negative No. 12
Bcl-2 negative No. 13
Bcl-2 negative No. 14
Bcl-2 negative No. 15
Cells/mm
Weeks
0
2
4
6
8
10

Figure S3

Figure S3. The number of immature epidermal melanocytes for each patient in the MITF and Bcl-2 staining (related to main Figure 2 and Table 1)

Number of cells per millimeter in the basal layer for each patient (n = 15 patients). MITF-negative cells (●) and Bcl-2–negative cells (▲) morphologically characterized as melanocytes.

S4

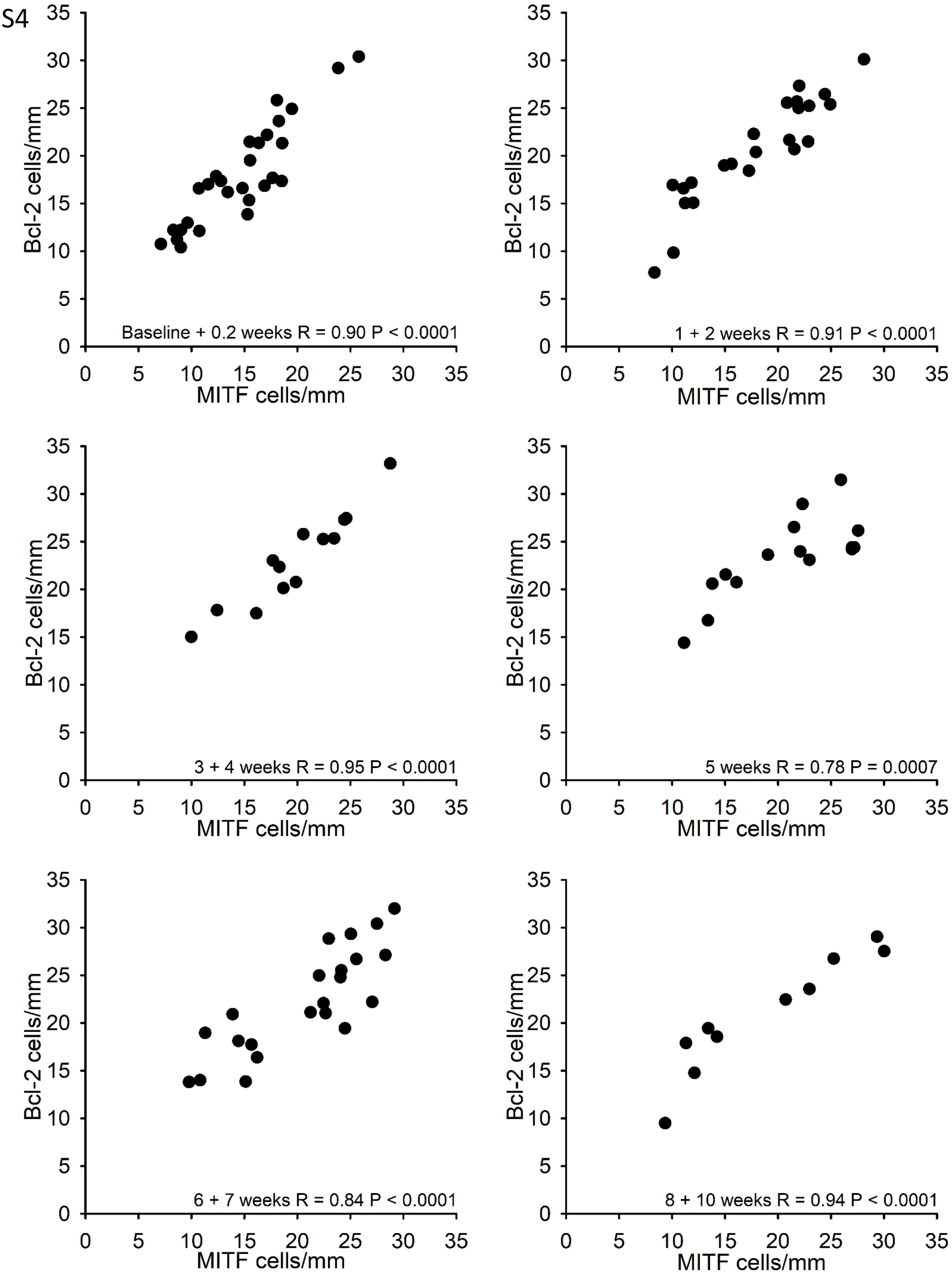

Baseline + 0.2 weeks R = 0.90 P < 0.0001
1 + 2 weeks R = 0.91 P < 0.0001
3 + 4 weeks R = 0.95 P < 0.0001
5 weeks R = 0.78 P = 0.0007
6 + 7 weeks R = 0.84 P < 0.0001
8 + 10 weeks R = 0.94 P < 0.0001
Bcl-2 cells/mm
MITF cells/mm
0
5
10
15
20
25
30
35

Figure S4

Figure S4. Individual expressions of MITF and Bcl-2 in interfollicular melanocytes (related to main Figure 2 and Table 1)

Correlation between MITF and Bcl-2: pre-, during, and post-radiotherapy. Cells/mm for each patient at a certain time point.

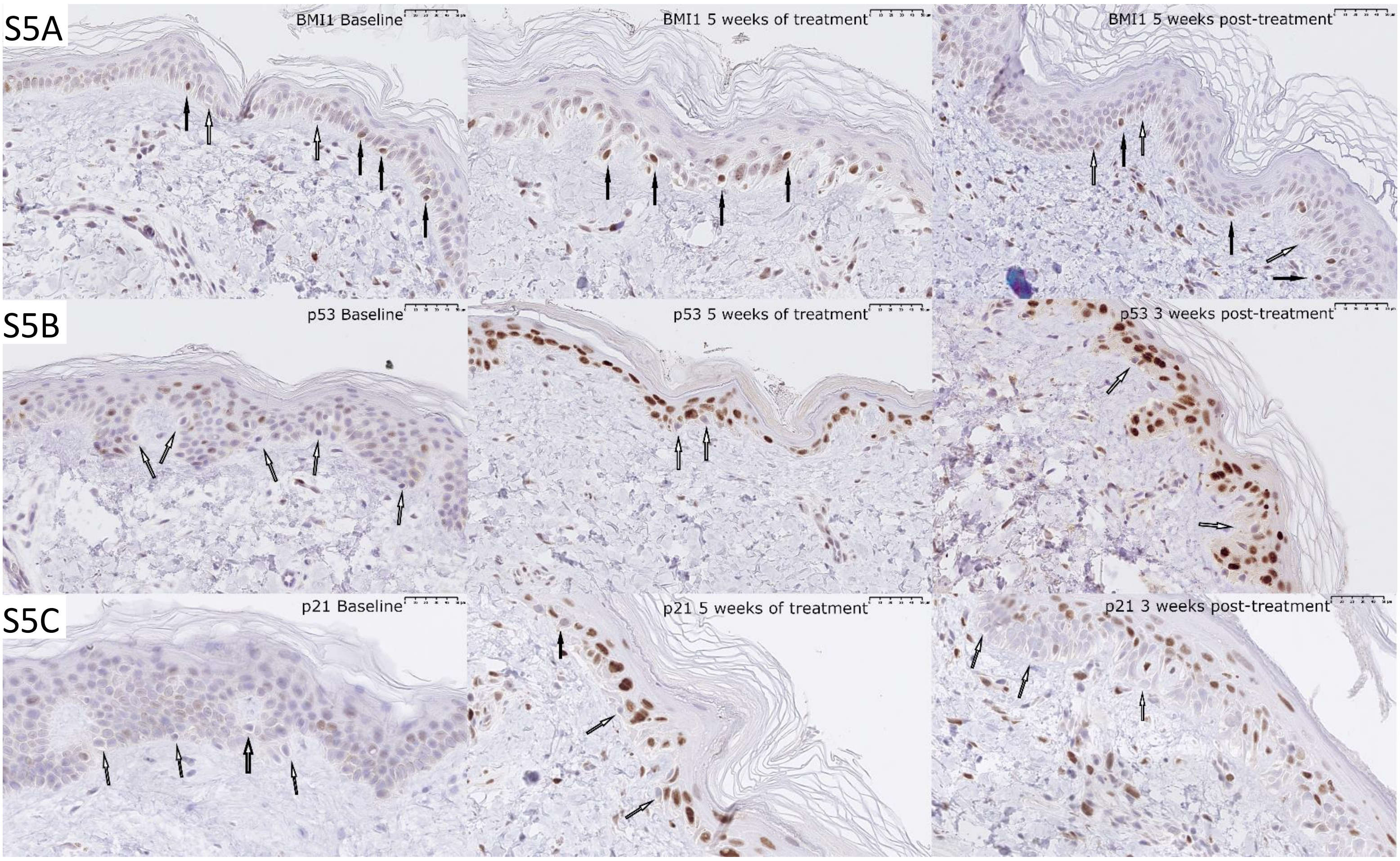

Figure S5. Interfollicular melanocytes express nuclear BMI1 but not p53 and rarely p21 in the nucleus (related to main figure 3, 6 and 7)

Staining for BMI1 (S5A), p53 (S5B) and p21 (S5C) in skin biopsies before radiotherapy (RT), after 5 weeks of treatment, and at 3 or 5 weeks post-treatment. Black arrows indicate examples of stained melanocytes, and the white arrows indicate unstained melanocytes. Of note the figure image does not reflect the cell membranes as clearly as in 1000× magnification.

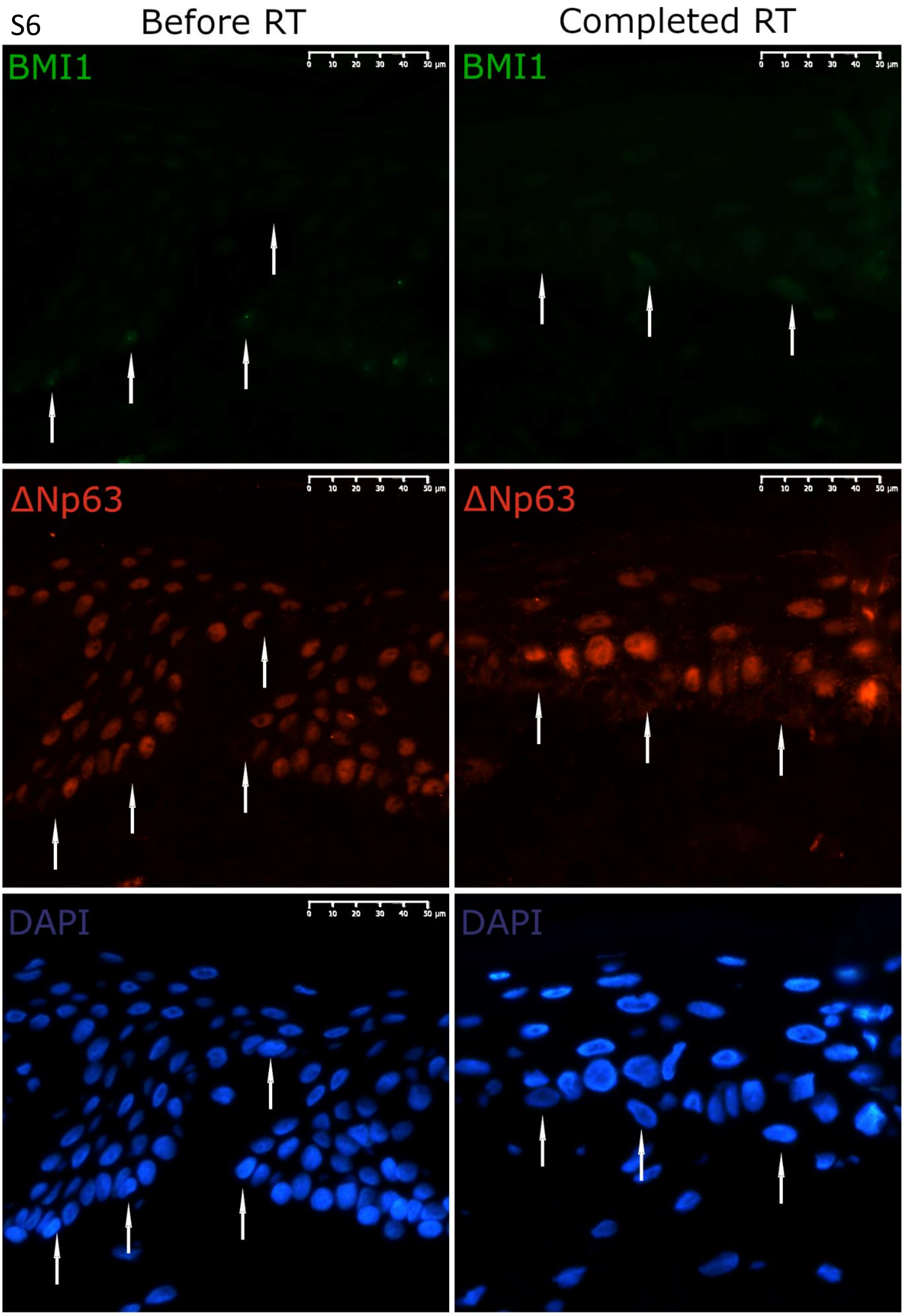
S6
Before RT
Completed RT
BMI1
BMI1
ΔNp63
ΔNp63
DAPI
DAPI
0 10 20 30 40 50 μm

Figure S6

Figure S6. BMI1-positive cells belong to the melanocyte lineage (related to main Figure 3)

Double staining of BMI1 and ΔNp63. Staining patterns in biopsies acquired before treatment are compared to staining at the end of 5 weeks of treatment. Cells in the melanocyte lineage are negative for ΔNp63; this staining visualizes the melanocyte population throughout radiotherapy. Arrows in the left column respectively indicate three BMI1-positive melanocytes and one BMI1-negative melanocyte in the basal layer prior to ionizing radiation (IR) exposure. Two BMI1-positive melanocytes and one BMI1-negative melanocytes are highlighted by arrows in the right column, illustrating the staining pattern after completed IR exposure.

S7

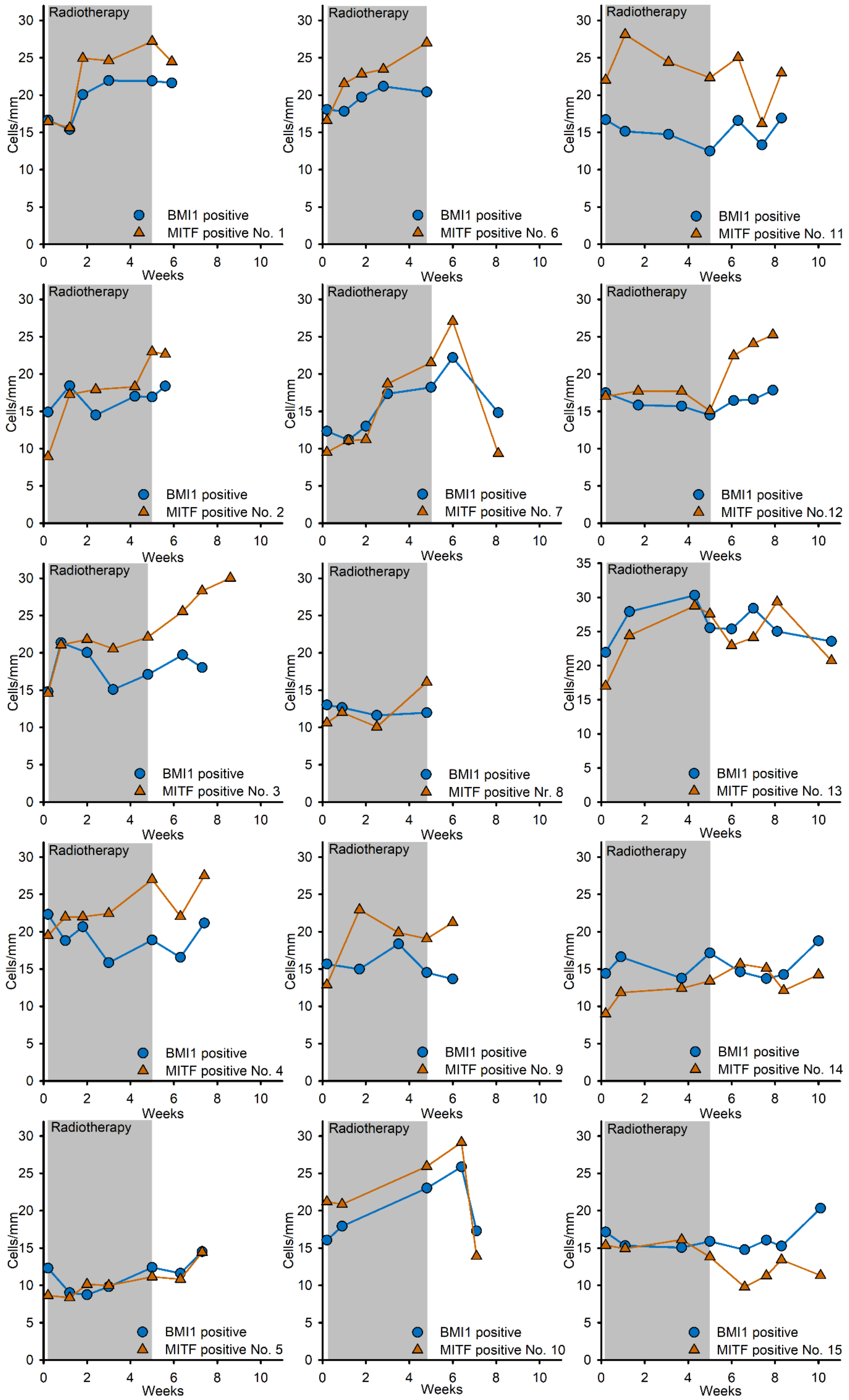
Radiotherapy
Cells/mm
Weeks
BMI1 positive
MITF positive No. 1
MITF positive No. 6
MITF positive No. 11
MITF positive No. 2
MITF positive No. 7
MITF positive No.12
MITF positive No. 3
MITF positive Nr. 8
MITF positive No. 13
MITF positive No. 4
MITF positive No. 9
MITF positive No. 14
MITF positive No. 5
MITF positive No. 10
MITF positive No. 15

Figure S7

Figure S7. The number of interfollicular melanocytes in each individual patient expressing MITF and BMI1 (related to main Figure 2, 3 and Table 1)

Number of epidermal melanocytes per millimeter in the basal layer for each patient (n = 15 patients) expressing MITF (▲) and BMI1 (●).

S8

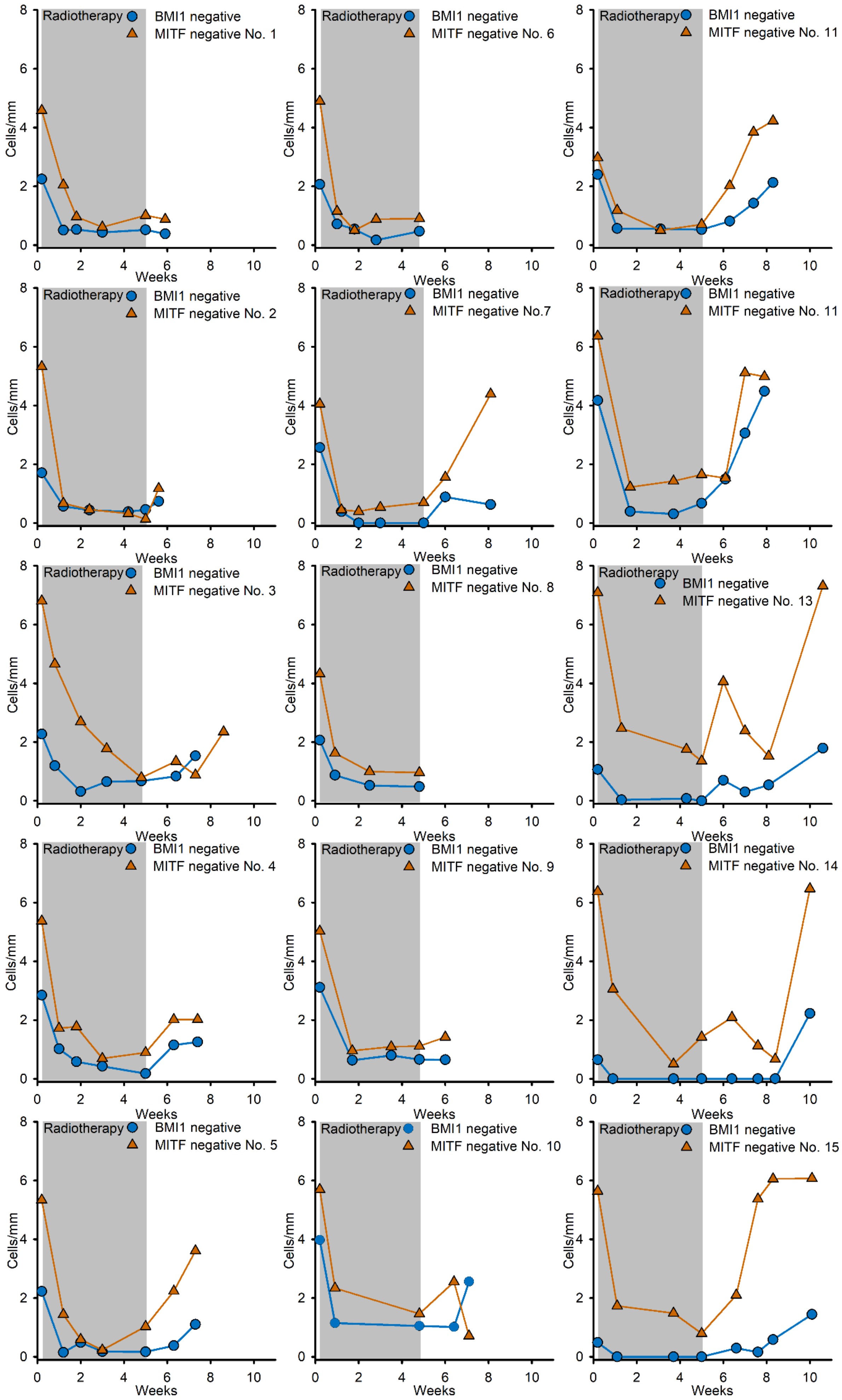
Radiotherapy
BMI1 negative
MITF negative No. 1
MITF negative No. 6
MITF negative No. 11
MITF negative No. 2
MITF negative No.7
MITF negative No. 11
MITF negative No. 3
MITF negative No. 8
MITF negative No. 13
MITF negative No. 4
MITF negative No. 9
MITF negative No. 14
MITF negative No. 5
MITF negative No. 10
MITF negative No. 15
Cells/mm
Weeks

Figure S8

Figure S8. The number of immature epidermal melanocytes in each individual patient in MITF and BMI1 staining (related to main Figure 2, 3 and Table 1)

Number of epidermal melanocytes per millimeter in the basal layer for each patient (n = 15 patients), which are MITF-negative (▲) and BMI1–negative (●) and morphologically characterized as melanocytes.

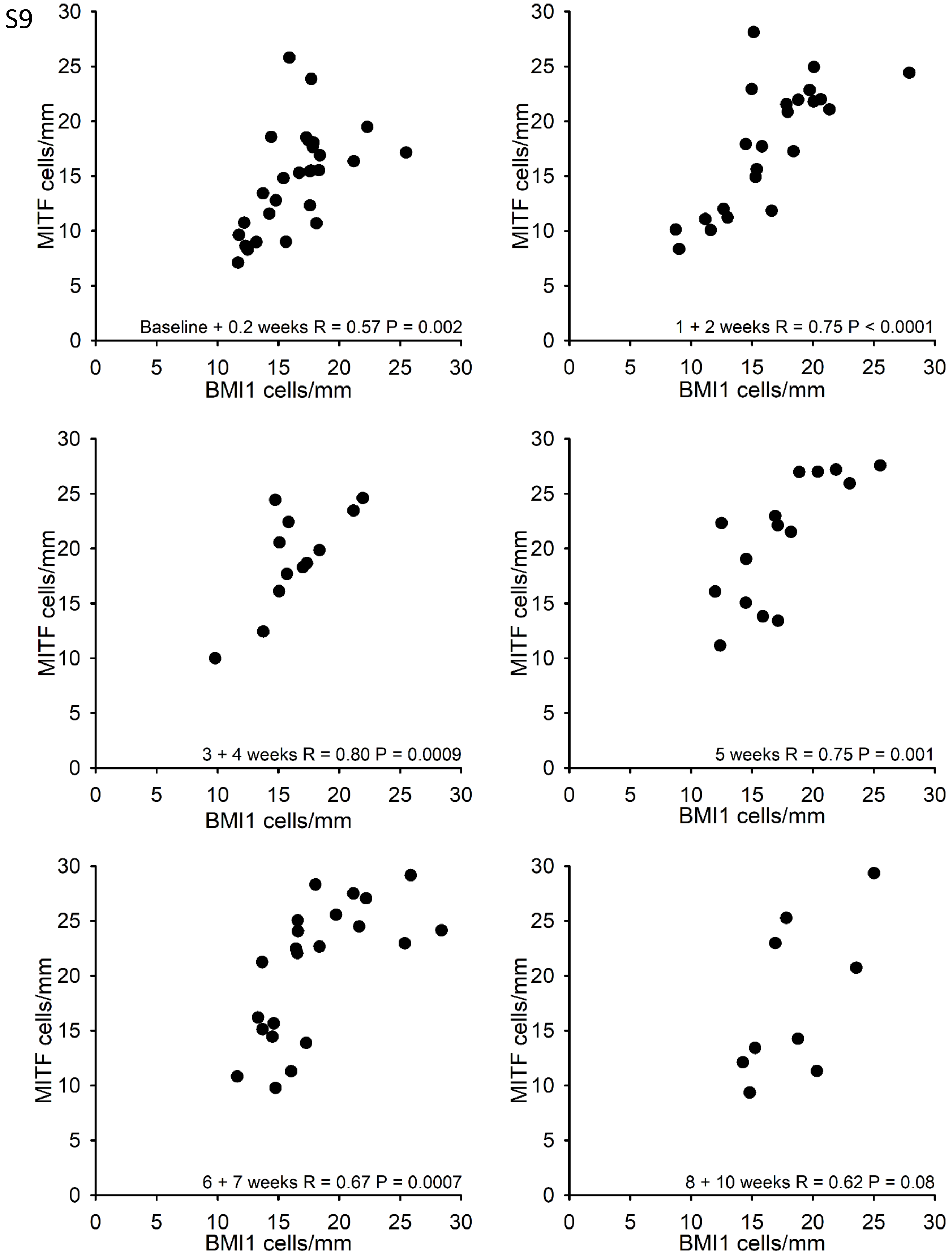
S9
MITF cells/mm
BMI1 cells/mm
Baseline + 0.2 weeks R = 0.57 P = 0.002
1 + 2 weeks R = 0.75 P < 0.0001
3 + 4 weeks R = 0.80 P = 0.0009
5 weeks R = 0.75 P = 0.001
6 + 7 weeks R = 0.67 P = 0.0007
8 + 10 weeks R = 0.62 P = 0.08

Figure S9

Figure S9. Individual expressions of MITF and BMI1 in interfollicular melanocytes (related to main Figure 2, 3 and Table 1)

Correlation between BMI1 and MITF: pre-, during, and post-radiotherapy. Cells/mm for each patient at a certain time point.

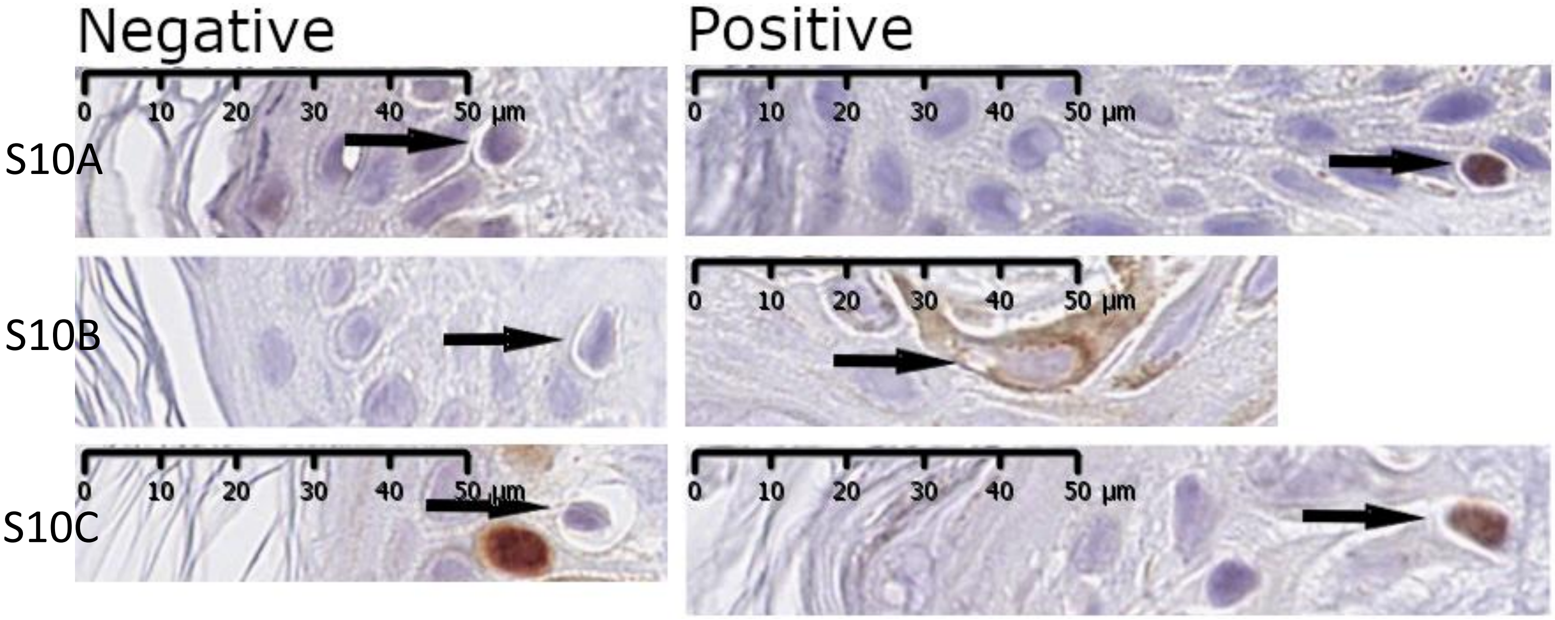

Figure S10. Cell cycle progression markers among interfollicular melanocytes (related to main Figure 5)

Immunostaining for cyclin A (S10A), cyclin B1 (S10B), and pRb (S10C) in skin biopsies demonstrating negative and positive melanocytes. Black arrows indicate examples of negative and positive melanocytes. Of note the figure image does not reflect the cell membranes as clearly as in 1000× magnification.

Table S1. Total number of epidermal melanocytes per millimeter and total number of melanocytes counted for each marker. (related to main Figure 2, 3 and Table 1)

**Staining**	**Time in weeks** | *number of biopsies* | number of patients										
	**0**|*30*|15	**0.2**|*13*|13	**1**|*19*|13	**2**|*13*|10	**3**|*10*|8	**4**|*9*|5	**5**|*15*|15	**6**|*13*|13	**7**|*9*|9	**8**|*7*|7	**10**|*3*|3
MITF	19.3±1.1	20.5±1.4	19.5±1.7	19.2±1.9	21.3±1.7	19.8±2.9	21.8±1.5	23.4±1.6	22.2±2.1	23.8±3.2	22.1±3.2
	4698	2582	2862	1811	1607	1409	2399	2266	1605	1461	464
Bcl-2	20.9±1.1	24.4±1.5	22.4±1.9	22,0±1.9	26.1±2.3	25.8±2.9	25.7±1.5	23.8±1.5	24.4±1.8	24.8±3.5	26.6±4.1
	4357	1941	2944	1855	1651	1329	2289	2046	1396	1272	297
BMI1	18.5±0.9	18.9±0.9	17.3±1.3	16.4±1.3	17.2±1.4	18.5±3.0	17.8±1.0	19.0±1.3	18.9±1.6	18.7±1.8	22.7±1.3
	3300	1473	2167	1186	1069	987	1664	1437	1051	797	263

Data are presented as mean and standard error of the mean.
